# Mapping antimicrobials in plant lineages

**DOI:** 10.3389/fpls.2026.1936509

**Published:** 2026-09-03

**Authors:** Gabin Thierry M. Bitchagno, Paula Coates, Erin Garcia, Sohini Bhatia, Amorita Correia, Debbie Mulligan, Scott Bintrim, Monique S.J. Simmonds

**Affiliations:** 1 Royal Botanic Gardens, London, United Kingdom; 2 The Procter & Gamble Company, Reading, United Kingdom; 3 Mason Business Center, The Procter & Gamble Company, Mason, OH, United States

**Keywords:** metabolomics, natural antimicrobials, phylogeny, plants, preservatives

## Abstract

Plant species with antimicrobial activity occur across many angiosperm families, yet the factors driving the diversity of bioactive compounds remain unclear. This study investigates the antimicrobial activity of 285 flowering plants representing 65 families and 131 genera across lineages reputed to exhibit antimicrobial potential. Over 462 plant extracts were screened against *Staphylococcus aureus*, *Escherichia coli* and *Aspergillus brasiliensis*. Active extracts were fractionated and metabolomically profiled using LC-MS and NMR to identify chemical drivers and their phylogenetic distribution. About 37% of extracts, including preliminary and serial extracts, inhibited *S. aureus* and 4% inhibited *E. coli* (MIC ≤ 1000 µg/mL). Hexane and EtOAc extracts showed the highest activity against *S. aureus* while 60%MeOH extracts showed limited activity against *E. coli*. The lowest MIC values were <1.95 and 125 µg/mL, respectively, and no antifungal activity was detected. Active extracts mainly contained phenols or diterpenes with characteristic pharmacophores. Phylogenetic comparison revealed antimicrobial hotspots, highlighting plants as promising sources of anti-*S. aureus* compounds but limited leads against *E. coli* or *A. brasiliensis*.

## Introduction

Natural antimicrobials mostly refer to plant- and fungi-derived preparations capable of inhibiting microbial growth. These include crude extracts, refined fractions, and isolated pure compounds sourced from nature. The subject continues to capture global interest, largely due to the complex interplay between microbes and human health, which has contributed to one of the most pressing health threats of this century, antimicrobial resistance (AMR). Natural products are recognized as a key part to the solution, having served as the foundation for over half of the antimicrobial drugs approved to date by the FDA and its European counterpart (Domingo-Fernández et al., 2024). Plants represent a particularly attractive resource, offering both a high diversity of species and substantial biomass availability. Although microorganisms offer an even greater diversity and therefore a higher discovery potential, it remains difficult to find sources with sufficient biomass to support industrial-scale production (Liu et al., 2017).

Interest in natural antimicrobials across many sectors is driven by an increasing consumer demand for greener, sustainable, and environmentally friendly products (Li et al., 2024). As preservatives, antimicrobials are of critical importance in all industries, as the vast majority of products coming off production lines require chemical agents to ensure their stability and longevity on store shelves (Fan et al., 2018). Both bacteria and fungi bear the responsibility for this degradation (Sionek et al., 2024). Their susceptibility to antimicrobials happens through multiple pathways. Indeed, Gram-positive bacteria have a single, thick, cell wall composed primarily of peptidoglycans, whereas Gram-negative bacteria and fungi feature a double-layered cell wall (Long and Li, 2024). The simple mechanism by which substances impair bacteria is through the hydrolysis or disruption of cell walls. In the case of Gram-positive bacteria, the mesh-like structure of peptidoglycans allows compounds to pass through the pores of the membrane. In contrast, the lipopolysaccharide layer of Gram-negative bacteria or that of glycosylated proteins in fungi prevent this, shielding the cell and regulating the transport of molecules and nutrients across the membrane (Ivanov et al., 2022; Long and Li, 2024). That leaves Gram-positive bacteria relatively more susceptible to antibiotics than Gram-negative bacteria and fungi.

That susceptibility extends also to encompass plant antimicrobials. Numerous chemical scaffolds have been discovered as potential plant-based leads against microbial infections. Putting aside alkaloids of which many are also known to be cytotoxic, promising candidates include prenylated phenolics, terpenes, and alkylphenols (Arora et al., 2023). These compounds are considered widespread across different flowering plant families, although their links to antimicrobial activity remain poorly documented. The present study contributes to our understanding of plants with anti-microbial activity by investigating the activity and compounds associated with this activity in 285 species of plants from 131 genera and 65 families. Their activity was mapped onto the phylogeny of the flowering plants of these 285 species, the team has already published data on the antibacterial activity of 13 species (Bitchagno et al., 2025a, b; 2026). Data from these species has been included as they contribute to the mapping onto the phylogeny of the flowering plants.

## Materials and methods

### General experimental procedure

LC–MS grade solvents (acetonitrile, methanol) and formic acid were obtained from Fisher Scientific (Loughborough, UK) and milliQ water was used for HPLC and LC-MS analysis. NMR spectra were acquired on a Bruker Avance-III (^1^H NMR: 400 MHz and ^13^C NMR: 100.1 MHz) spectrometer equipped with a 5 mm cryoprobe. Chemical shifts were referenced to residual solvent signals and reported in parts per million (ppm). Spectra were processed using Bruker NMR academic Topspin software. Mass spectra were collected on a Orbitrap Exploris mass spectrometer, equipped with an Orbitrap Exploris 120 with a heated ESI source (Thermo Scientific, Germany), acquired in both negative and positive modes with a resolution of 60,000 over *m/z* 125–1800 under various acquisition parameters like source voltages, sheath gas, auxiliary gas, sweep gas and capillary temperature set to 2.5 kV (negative mode) and 3.5 kV (positive mode), 50 (arbitrary units), 10 (arbitrary units), 1 (arbitrary units) and 350 °C, respectively. Automatic MS–MS fragmentation was performed on top four ions of the TIC using an isolation width of *m/z* 2. High-energy C-trap dissociation with a normalized collision energy of 40 and an activation time of 0.1 ms was served to fragment ions. The MS unit is interfaced with a Vanquish Core UHPLC system, which includes a Vanquish diode array detector (VH-D10) operating at four wavelengths: 210 nm, 254 nm, 300 nm, and 366 nm. Samples are injected using an autosampler maintained at 30 °C, while the analytical column ACQUITY Premier CSH C18 1.7 µm, 2.1 x 150 mm (Waters Corporation, Milford, MA, USA) is kept at a constant temperature of 35 °C. The injection volume for each sample was 1 *µ*L at a flowrate of 200 µL/min and the gradient was an increase of acetonitrile (B) in water (A) (0–5 min, 10% B; 5–20 min, 10 to 100% B, 20 to 30 min, 100% B and 30 to 35 min, 10% B). Collected data were inspected using Xcalibur v. 4.2.47 (Thermo Fisher Scientific). Chemical profiling of extracts was conducted on a Biotage^®^ Isolera One system for splitting extracts into small fractions and a Waters Alliance 2695 HPLC system for isolation of compounds. A reversed-phase Discovery HS C-18 column (5 *μ*m, 10 mm × 250 mm i.d., Supelco, UK) maintained at 35 °C served in compounds isolation and purification over gradient of acetonitrile+0.1% formic acid (A) and water (B).

### Plant material

The plant materials were mostly collected from the living collection at RBG Kew but some were provided by either Sir Harold Hilliers Gardens, Hampshire UK or trade suppliers (see **Supplementary Materials** for details). The names and identities of species are verified and recorded on an internal database (Bramhs v8.5.2). Plants from RBG Kew are usually given an accession number that records where they come from and any restriction on their use. Some plants used in ornamental displays or those considered as weeds are not given an accession number. When material is subject to biochemical analysis it is given a unique BI number for tracking purposes. The materials from RBG Kew and Sir Harold Hilliers Gardens were collected fresh, freeze-dried, milled to fine powders, and kept in the dark until further use. Material from trade suppliers were either supplied as an extract or dried.

### Extraction for preliminary bioassays

The dried material was milled using a laboratory mill and subjected to a preliminary extraction with two repeats of 100 mg of plant material suspended in 2 mL of 20.80 (*v/v*) water/methanol (eventually ethanol, when required), vortexed vigorously for 10s, kept in solvent 16 hours at room temperature (or eventually heated in a water bath kept at 50 °C for 10 min), and centrifuged at 15,600 rpm for 10 min. Part of the supernatant (1.5 mL) was transferred to a clean flask and dried using a GeneVac concentrator (Genevac, Suffolk, UK). If at least 10 mg of extract were recovered, the samples were dissolved in DMSO to constitute a solution of 20 mg/mL and sent for bioassays. Otherwise, the process was repeated to bulk the extract to at least 10 mg (Kisiriko et al., 2024, 2025).

### Serial extraction and purification

Active plant materials with an MIC ≤ 1000 ppm were collected (about 500 g of fresh material) from the living collection, freeze dried, milled and serial extracted in solvents with increasing-polarities starting with *n*-hexane, then EtOAc and 60% MeOH affording dried extracts. Following bioassay results, the most active extracts were each split into small fractions on the Biotage Isolera One system using a stepwise gradient of EtOAc (B) in hexane (A) (3CV, 100% A; 3CV, 10% B, 3CV, 20% B, 3CV, 30% B; 3CV, 40% B, 3CV, 50% B and 3CV, 100% B). For most hexane extracts, the gradient applied during the fractionation, on the same system, was a stepwise increase of isopropanol (B) in MeOH (A) (3CV, 5% B; 3CV, 10% B, 3CV, 20% B, 3CV, 30% B; 3CV, 40% B, 3CV, 50% B and 3CV, 100% B) while the 60% MeOH fractionation was done, still on the same Biotage system, but with a gradient of MeOH (B) in deionized water (A) (3CV, 5% B; 3CV, 10% B, 3CV, 20% B, 3CV, 30% B; 3CV, 40% B, 3CV, 50% B and 3CV, 100% B). Both hexane and 60% MeOH extracts fractionation was done on a SNAP Ultra C18–60 g cartridge from Biotage with a flowrate of 30 mL/min, whereas the EtOAc extract fractionation was achieved on various silica cartridge, also from Biotage in a ratio of 1g of extract for 10g of cartridge silica mass. The flowrates were usually defined automatically by the system. The elutions were collected into tubes of 20 mL each and into small fractions (BI Number_E1, 2, 3, …, n) reflecting the separation showed on the peak chromatogram of each run. The fractions were dried down on a Rotary evaporator, dissolved in CDCl_3_ and submitted to ^1^H NMR then to 2D NMR analysis for chemical profiling. Each NMR dataset was carefully inspected to identify patterns leading to the profiling of major, and sometimes minor, constituents of the fraction. A solution of 1 mg/mL of each fraction was also prepared and analyzed by LC-MS for confirmation of the structures and annotation of the extracts (Bitchagno et al., 2025c).

### Antimicrobial assays

Minimum Inhibitory Concentration (MIC) assays were used to determine the minimum concentration of an active required to inhibit half of the growth of microorganisms. Compounds were evaluated against a range of organisms including the Gram-positive bacterium, *Staphylococcus aureus* ATCC6538, the Gram-negative strain, *Eschierichia coli* ATCC8739 and the mold *Aspergillus brasiliensis* ATCC16404 (Bitchagno et al., 2025a, b, 2026). Microbial solutions were prepared in saline (bacteria) and saline with tween (mold) and adjusted turbidometrically to a target concentration of 10^7^–10^8^ CFU/mL. This inoculum solution was further diluted in Tryptone Soy broth (*S. aureus* and *E. coli*) or Soya Dextrose broth (*A. brasiliensis*) to achieve a final inoculum level of approximately 10^5^ CFU/mL assay use. A stock 96-well plate of the extracts were prepared in dimethyl sulfoxide (DMSO) at a concentration of 20 mg/mL and serially diluted in DMSO. For each test plate, 5 *µ*L of each dilution (each well from the stock plate) was transferred to a new test plate and 195 *µ*L of inoculum in broth was added to each well. The *S. aureus* and *E. coli* plates were incubated on an orbital sharker for 18 ± 2 hrs at 32.5 °C. The *A. brasiliensis* plates were inoculated at 20-25 °C for 5 days. After incubation, the plates were visually assessed, and the MICs were determined as the most diluted well with reduced growth (~50%) compared to growth control. Piroctone olamine for antibacterial, (MIC 15.6 *µ*g/mL or 52.3 *µ*M vs *S. aureus*) and zinc pyrithione for antifungal (MIC 0.98 *µ*g/mL or 3.1 *µ*M vs *C. albicans*) activities were used as positive controls.

## Results

### Selection of plants and strategy

**Figure 1:**
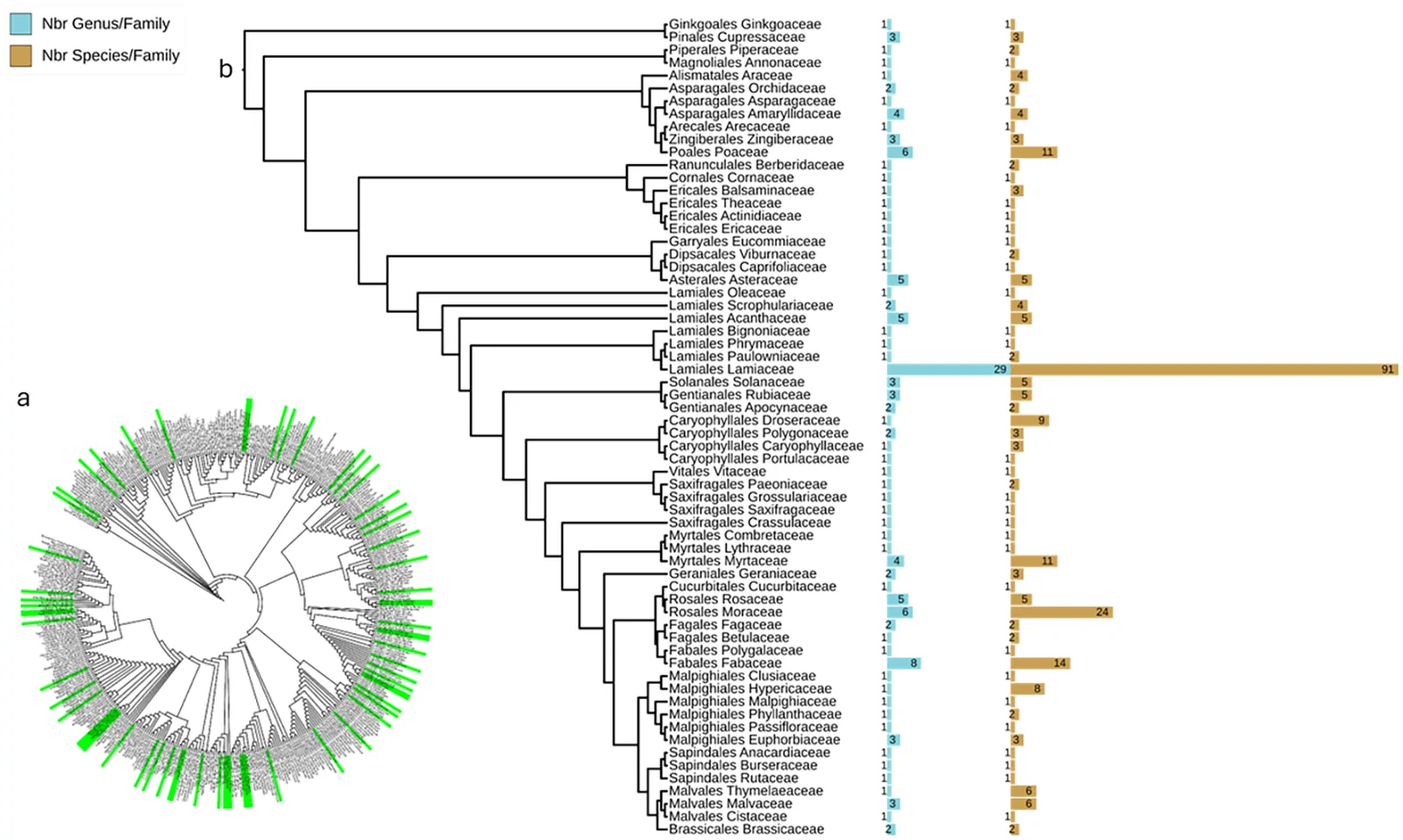
**(a)** Angiosperm tree of life (Baker et al., 2022) with highlighted families of plants used in the study. **(b)** Tree of families used in the study as represented in the phylogeny of angiosperms. Bar charts representing the numbers of genera and species per family of plants examined.

The overall samples represented 65 plant families belonging to different orders spread across the angiosperm tree of life (**Figure 1a**). The selection of plants followed a structured methodology in which plants rich in alkaloids were excluded, because they are usually poisonous or toxic (Akinboye et al., 2023). Rather, attention was placed on plant species reported in the literature to exhibit antibacterial activity, regardless of their reported MIC values (**Supplementary Table S1**). Plants were also selected for their ethnopharmacological indications in traditional medicine worldwide including those used to treat infectious diseases. Some species growing at Kew Gardens were opportunistically included in this study because they were being pruned. **Supplementary Table S1** summarizes the plants used in the study.

The 285 species of plants studied belong to 131 genera from 65 plant families. Some families were represented by only a single genus, and some genera by only one species, such as *Ginkgo biloba*, which is also the sole surviving species of its genus. In contrast, certain families, such as Lamiaceae, Moraceae, and Fabaceae, included multiple genera and a diversity of species (**Figure 1b**). For several plant families, enough genera and species were included to observe how activity might be distributed within the family. This was the case for Lamiaceae (29 genera, 91 species), Moraceae (5 genera, 24 species), Myrtaceae (4 genera, 11 species), and Fabaceae (8 genera, 14 species). In other instances, the study included multiple species within a single genus, allowing the comparison of activity within that genus. Examples include *Drosera* (9 species), *Eucalyptus* (6 species), *Coleus* (10 species), *Phyllostachys* (4 species), *Hypericum* (7 species), and *Daphne* (5 species).

### Preliminary bioassays

**Figure 2:**
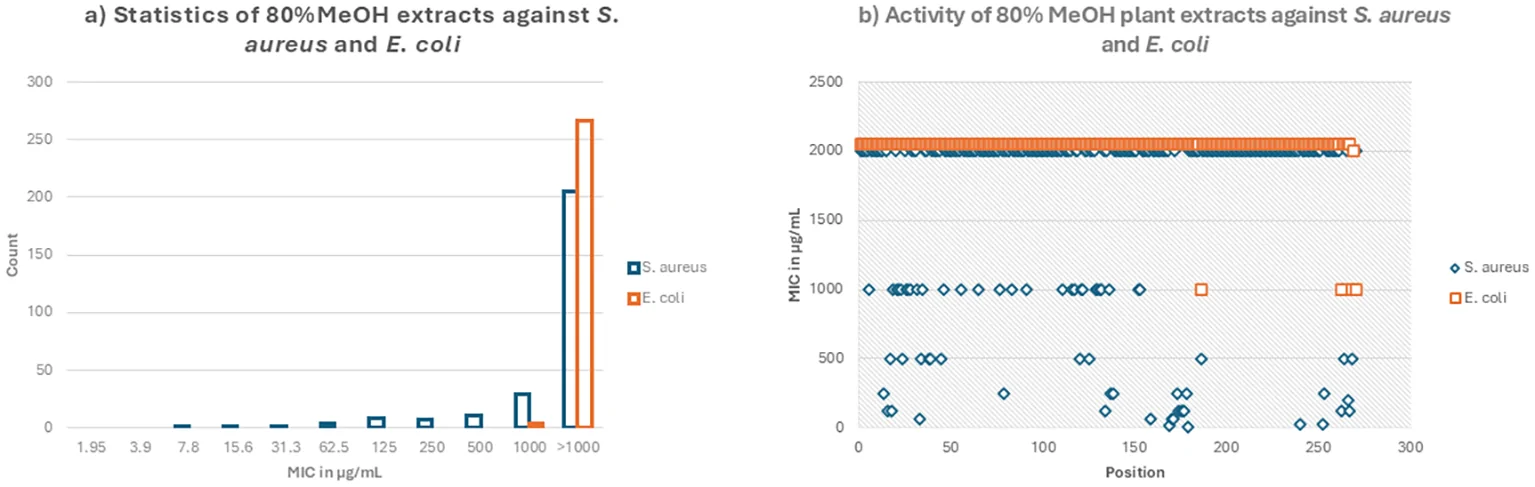
**(a)** Bar chart displaying the numbers of preliminary extracts tested against *S. aureus* and *E. coli* per concentration **(b)** Scatter plot of preliminary extracts tested against *S. aureus* and *E. coli* showing the distributive performance of preliminary extracts (their likelihood to perform at a given MIC value).

Extracts from each of the 285 plants were prepared in 80% methanol in water and screened for antimicrobial activity against *Staphylococcus aureus*, *Escherichia coli* and *Aspergillus brasiliensis*. Of the 285 extracts generated, 66 (23%) exhibited activity against *S. aureus*, with MICs ranging from 7.8 µg/mL to 1000 µg/mL (**Figure 2a**). Only one extract was active at 7.8 µg/mL another at 15.6 µg/mL, two at 31.3 µg/mL, four at 62.5 µg/mL, nine at 125 µg/mL, seven at 250 µg/mL, eleven at 500 µg/mL, and twenty-nine at 1000 µg/mL (**Figure 2b**). On the other hand, only four extracts had MIC values at 1000 µg/mL against *E. coli*, and nothing at lower concentrations. These *E. coli* active extracts corresponded to three *Drosera* species and one *Quercus* species (**Figure 2b**). Interestingly, all extracts that were active against *E. coli* also showed activity against *S. aureus*.

### Serial extract bioassays

**Figure 3:**
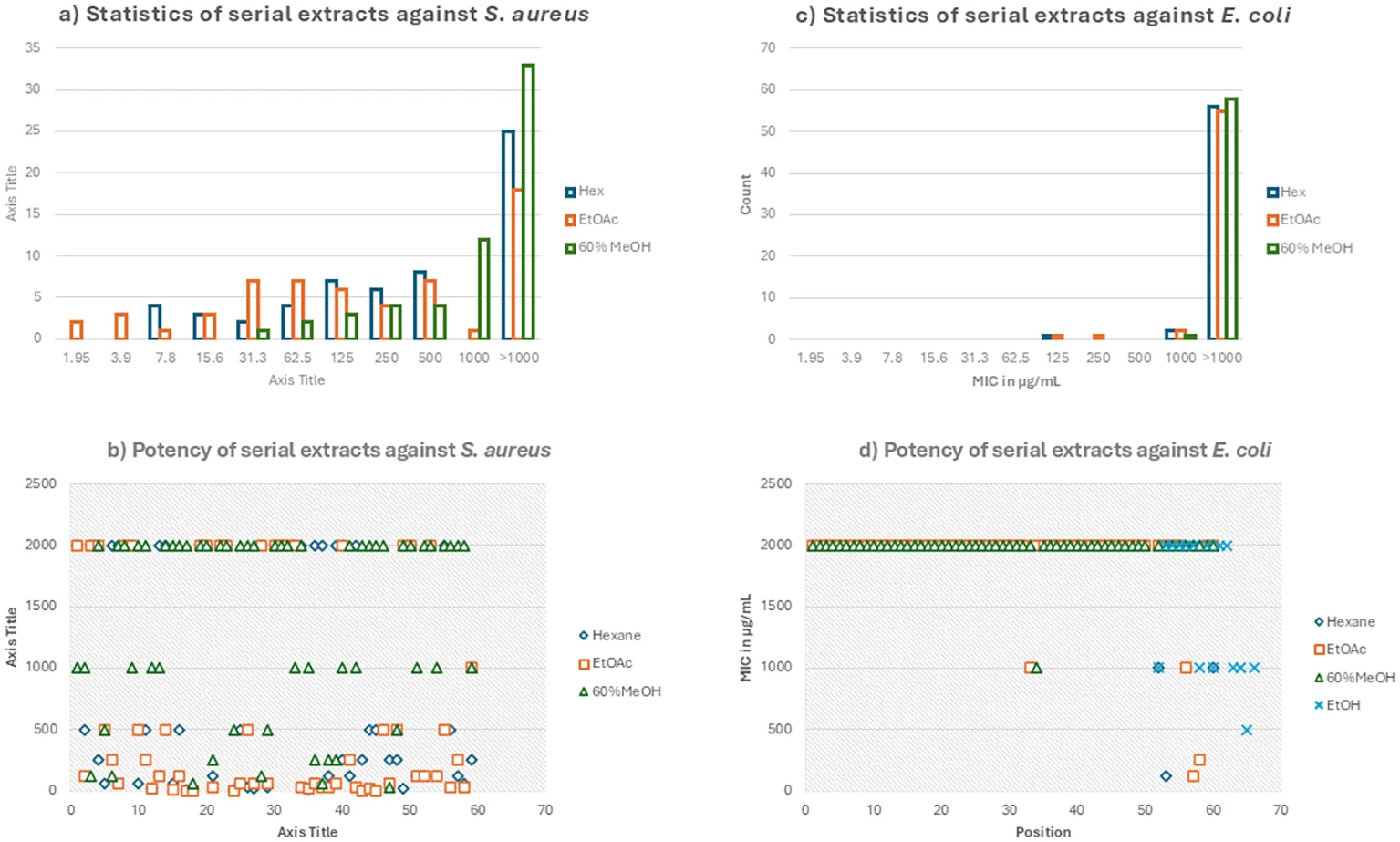
Analytics of bioassays turnout of serial plant extracts: **(a)** Bar chart displaying the numbers of samples tested against *S. aureus* per concentration and per extract types, **(b)** Scatter plot of serial extracts tested against *S. aureus* showing the distributive performance of Hex, EtOAc and 60%MeOH extracts (their likelihood to perform at a given MIC value), **(c)** Bar chart displaying the number of samples tested against *E. coli* per concentration and per extract types, **(d)** Scatter plot of serial extracts tested against *E. coli* showing the distributive performance of Hex, EtOAc and 60%MeOH extracts (their likelihood to perform at a given MIC value).

Following the preliminary bioassays, 59 of the 66 active extracts were serially extracted in *n*-hexane, ethyl acetate (EtOAc), and 60% methanol in water (60%MeOH). Each extract was evaluated for its antimicrobial activity against the same pathogens. As observed in the preliminary assays, the fungal strain *A. brasiliensis* proved resistance to all extracts, whether polar or non-polar. In contrast, most extracts tested against *S. aureus* exhibited moderate to strong activity (**Figures 3a, b**). Indeed, approximately 39% of the EtOAc extracts showed potent activity, with a MIC ≤ 62.5 µg/mL, compared to 22% of hexane extracts and only 5% of the polar 60%MeOH extracts. Conversely, 29% of EtOAc, 36% of hexane, and 19% of 60%MeOH extracts demonstrated moderate activity, with 62.5 µg/mL < MIC ≤ 500 µg/mL. Additionally, the extracts obtained with 60%MeOH had the highest proportion of weakly active samples, with 20% exhibiting a MIC of 1000 µg/mL, compared to only one extract from EtOAc and none from hexane (**Figure 3a**).

The EtOAc extracts of *Plectranthus coleoides* and *P. crassus* were the most active against *S. aureus*, with a MIC of 1.95 µg/mL, followed by the EtOAc extracts of *Maclura pomifera*, *Drosera venusta*, and *D. aliciae*, all exhibiting a MIC of 3.9 µg/mL. Other extracts also demonstrated potent activity, like the EtOAc extract of *Plectranthus hadiensis* and the hexane extracts of *P. coleoides*, *P. crassus*, *M. pomifera*, and *Eucalyptus darympleana*, each with a MIC of 7.8 µg/mL. The EtOAc extract of *E. darympleana* showed less activity (MIC = 15.6 µg/mL), as did the EtOAc extracts of *Plectranthus elegans* and *Drosera capensis*, along with the hexane extracts of *P. elegans*, *Dracontomelon dao*, and *Lonchocarpus costaricensis*. Extracts with a MIC of 31.3 µg/mL included the EtOAc extracts of *Paulownia fargesii*, *Eucalyptus gunnii*, *E. aggregata*, *E. pauciflora*, *Drosera dichrosepala*, *Becium grandiflorum*, and *Aeollanthus densiflorus*, as well as the hexane extracts of *Lupinus arboreus* (leaves) and *Helichrysum arwae*. The group with the lowest, yet still potent, activity against *S. aureus* (MIC = 62.5 µg/mL) consisted of the EtOAc extracts of *Rabdosia rugova*, *L. arboreus* (stems), *E. perriniana*, *E. chapmaniana*, *Drosera affinis*, *H. arwae*, *L. costaricensis*, and the hexane extracts of *Salvia elegans*, *Plectranthus fructicosus*, *P. hadiensis*, and *Aeollanthus densiflorus*. **Figure 3b** illustrates the likelihood of EtOAc and Hex extracts to be potent against *S. aureus*, the summary of MIC values recorded is provided as supporting information (**Supplementary Table 2**).

As shown by the data from the preliminary assays, very few of these extracts showed activity against *E. coli* (**Figures 3c, d**), although 7% and 5% of the EtOAc and hexane extracts, respectively, showed moderate activity with MIC ≥ 125 µg/mL. The extracts exhibiting the most activity against *E. coli* were the hexane extract of *Drosera binata* and the EtOAc extract of *Drosera aliciae*, both with a MIC of 125 µg/mL. These were followed by the EtOAc extract of *Drosera macrantha* subsp. *macrantha* (MIC = 250 µg/mL) and the 60% MeOH extract of *Daphne tangutica* (MIC = 500 µg/mL). The hexane extracts of *Drosera gibsonii* and *Drosera platystigma*; the EtOAc extract of *Drosera capensis*; as well as the ethanol (EtOH) extracts of *D. gibsonii*, *D. platystigma*, *Daphne alpina*, and the leaves of *Daphne tangutica*, all showed a MIC of 1000 µg/mL against *E. coli*. Likewise, **Figure 3d** depicts the likelihood of EtOH, Hex and EtOAc extracts to be active against *E. coli*, the summary of MIC values recorded is also provided in **Supplementary Table 2**.

### Phylogeny propagation of the activity

**Figure 4:**
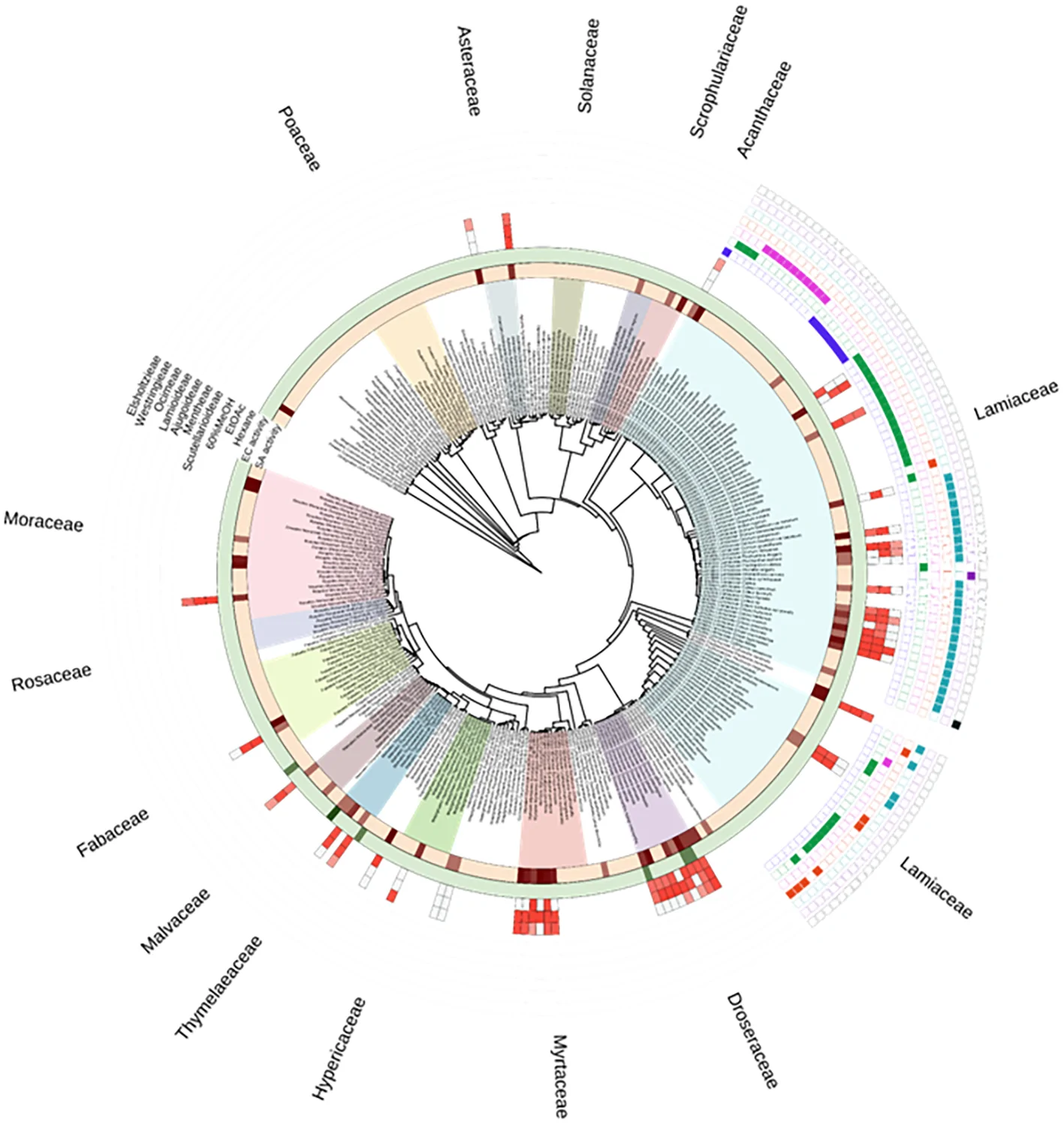
Species whose extracts showed activity against *S. aureus* and *E. coli*, have been mapped onto the molecular phylogeny published by Baker et al. (2022). Moving away from the center are rings of activity against *S. aureus* (brown) and *E. coli* (green) then heat map showing the activity of serial extracts and a second heat map mapping the classification of species of the Lamiaceae family onto the different tribes.subfamilies.

The 285 plant species were classified into their respective families and orders based on the molecular Tree of Life platform (Baker et al., 2022). These were then compiled into a phylogenetic tree of the plants used in the study (**Figure 4**), onto which the antimicrobial activity of extracts and fractions was mapped to provide an overview of how such activity is distributed across plant lineages. Similar to the overall plant selection, activity against *S. aureus* was dispersed across the entire phylogenetic tree, from early evolving lineages such as *Sequoia* spp. to more recently diverged groups like *Tilia* spp. or *Daphne* spp. Among these, the Lamiaceae emerged as the family with the highest number of active species (21 out of 91) against *S. aureus*. This family also showed the greatest diversity of active taxa, with representatives from multiple genera including *Coleus*, *Plectranthus*, *Aeollanthus*, *Isodon*, *Ocimum*, *Thymus*, *Scutellaria*, *Tinnea*, *Salvia*, and *Eriope*. However, none of the extracts of Lamiaceae species demonstrated activity against *E. coli*.

The Droseraceae family exhibited the highest proportion of active species against both *E. coli* and *S. aureus*, although limited to a single genus, *Drosera*. This family comprises only three living genera, with *Drosera* being the second most diverse after *Dionaea*. Among the nine species tested, seven inhibited *S. aureus* of which three also inhibited *E. coli*. Only two species of *Drosera* were inactive against both pathogens. A similar pattern was observed in the genus *Daphne* (Thymelaeaceae), where six species were tested, three showed activity against *S. aureus*, and two of these also inhibited *E. coli*. Within the order Malvales, species in the genera *Sterculia* and *Tilia* (Malvaceae), also demonstrated notable activity, against *S. aureus*.

None of the Rosaceae species tested exhibited antimicrobial activity. In contrast, some species from the Moraceae family, within the same Rosales order, displayed moderate to potent activity against *S. aureus*. They were a species of *Maclura*, two species of *Ficus* and three species from *Dorstenia* which exhibited activity against *S. aureus* with MIC of 500 µg/mL, 125-250 µg/mL and 15.6-1000 µg/mL, respectively. The remaining active species were sporadic, appearing as single representatives in families with more than one species in the study such as Scrophulariaceae, Hypericaceae, Fabaceae, and Acanthaceae.

### Chemical signatures of active groups of plants

**Figure 5:**
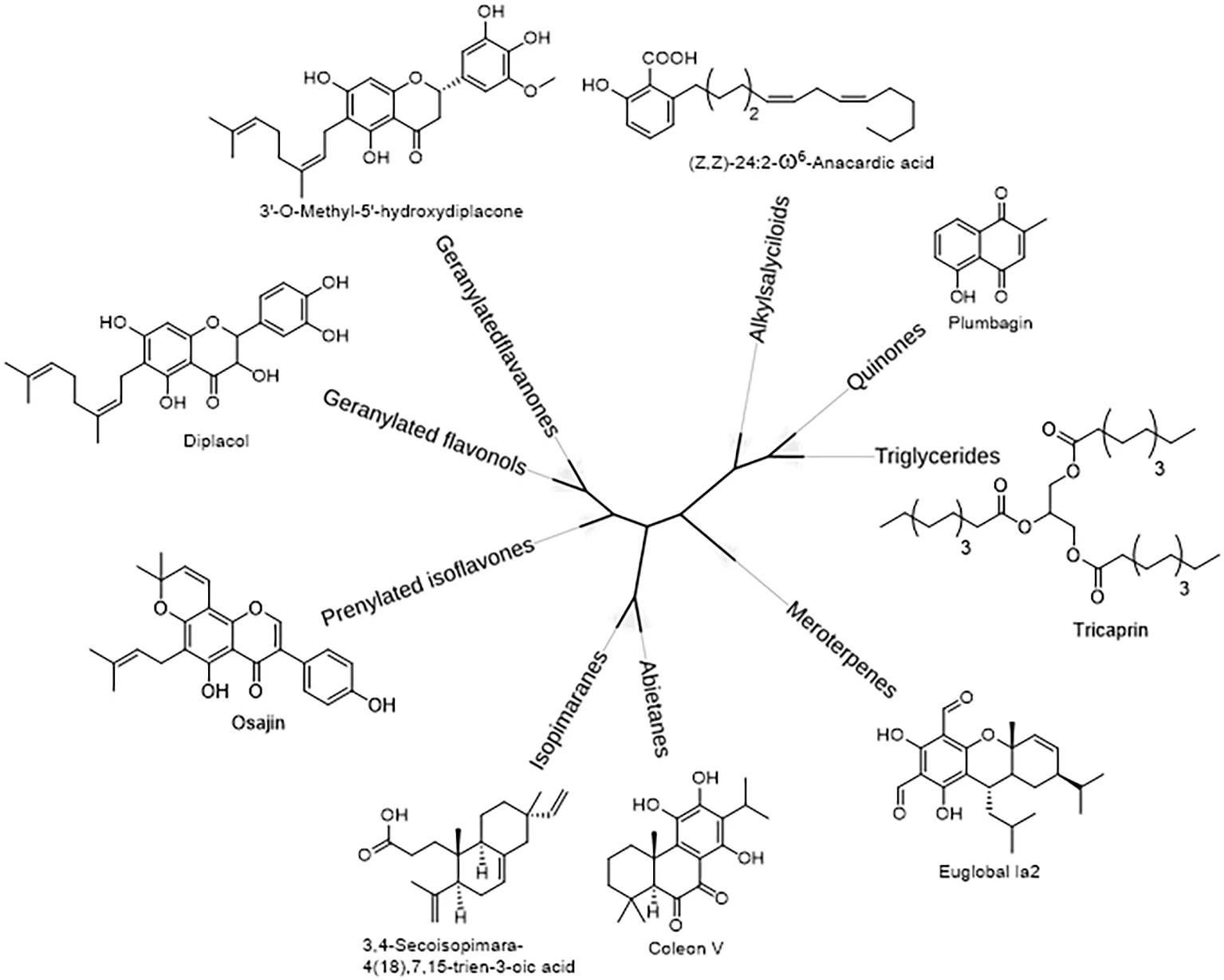
Diversity of compounds identified from the antimicrobial space of plant drivers against *S. aureus*.

Active serial extracts that exhibited MIC ≤ 62.5 µg/mL against *S. aureus* were successively flash chromatographed using a semi-automated system. The resulting fractions were evaluated for their activity against the same three pathogens. Active fractions were analyzed and profiled by 1D and 2D NMR, as well as by LC-MS.MS. Results suggested that six groups of compounds were responsible for the activity seen across all the plant extracts tested against *S. aureus*. They were diterpenes, prenylated flavonoids, meroterpenes, quinones, and alkylphenols (**Figure 5**).

Indeed, plants of the Lamiaceae family are well known for producing numerous classes of diterpenes, including isopimaranes and abietanes (Grayer et al., 2021; Bitchagno et al., 2025a, c, 2026). These two groups of diterpenes were found to be particularly widespread within the Lamiaceae in general. They were identified as major constituents in plants from the genera *Plectranthus*, *Aeollanthus*, *Coleus*, and *Salvia*. In addition to isopimaric acid, detected in *Aeollanthus densiflorus, A. buchnerianus* and *Sequoia sempervirens* with MIC = 7.8 µg/mL, the analysis also revealed that seco-isopimaranes, such as 3,4-secoisopimara-7,15-dien-3-oic acid isolated from *Salvia elegans*, also exhibit potent activity (MIC of 15.6 µg/mL) (Bitchagno et al., 2025a). Furthermore, species from closely related clades, such as *Plectranthus* and *Coleus*, showed a high occurrence of abietanes associated with some of the most potent antimicrobial activities (MIC of 1.95 µg/mL) of the study. This strong activity was particularly attributed to the presence of structural motifs such as *α*-ketoenol and *α*-diketone fragments, found in 11,12,14-trioxygenated phenolic abietanes or simply acylhydroquinone-type abietanes, such as coleons U and V, and their quinones and 14-deoxy derivatives (Bitchagno et al., 2026).

In addition, prenylated flavonoids found in species of the genera *Maclura* and *Paulownia* constituted another group of compounds that showed strong activity against *S. aureus* (Bitchagno et al., 2025b). Specifically, the isoflavones osajin, 6-prenylgenistein, and pomiferin were not only the major constituents of the plant fruits but also the constituents with the highest sensitivity against the Gram-positive bacterial strain with MIC values of 1.95, 7.8 and 15.6 µg/mL, respectively. On the other hand, various flavanones and flavanols derived from diplacol were isolated from *Paulownia fargesii*. The most active derivatives were diplacol, 3’-*O*-methyl-5’-hydroxydiplacone, diplacone, mimulone and 3’-*O*-methyl-5’-methyldiplacone with a MIC of 1.95 µg/mL, followed by 3’-*O*-methyldiplacol and 3’-*O*-methyldiplacone, which exhibited a MIC of 3.9 µg/mL. The activity of these derivatives seems not to depend on the oxidation at position C-3 as the susceptibility of *S. aureus* to 3’-*O*-methyldiplacol and 3’-*O*-methyldiplacone was identical. Rather, the potency of both groups of compounds was linked to the prenyl groups of their backbones.

Furthermore, the extracts and fractions obtained from *Eucalyptus* species that exhibited potent activity showed a relatively high concentration in polyformylphloroglucinols, a class of meroterpenes characteristic of this genus. They were mostly representated in the genus by the euglobals, particularly the stereoisomers euglobals Ia1 and Ia2. Fractions containing a mixture of these two compounds, though with a predominance in euglobal Ia2, demonstrated activity, with a MIC of 3.9 µg/mL. Fractions lacking these two compounds, but still containing other meroterpenes, showed slightly lower activity, with a MIC of 7.8 µg/mL. Among the compounds identified in these fractions (non-exhaustive list) were other euglobal derivatives, including euglobals IIb and IIc, as well as the meroterpene 4-*O*-demethylminiatone and the monoterpenes cryptomeridiol and its C-4 epimer.

Likewise, the strong activity against *S. aureus* recorded from *Dracontomelon dao* and its relative *Anacardium occidentale* were attributable to alkyl salicylates, produced in considerable amount in the Anacardiaceae family and represented primarily by anacardic acid. Alkylsalicylate derivatives isolated from *D. dao* contain two additional carbon atoms compared to those found in *A. occidentale*. Nevertheless, they all showed potent activity against *S. aureus*, with a MIC of 3.9 µg/mL. Moreover, in the most active species of the genus *Drosera*, a notable accumulation of methylated derivatives of ellagic acid was observed, along with quinones, primarily represented by plumbagin. These groups of compounds were complemented by glyceroglycolipids containing two aliphatic chains as well as by short-chain triglycerides like tricaprylin or tricaprin found in *D. macrantha subsp. macrantha* This combination of metabolites is proposed to be responsible for the observed activity, particularly against *S. aureus*, but also against *E. coli*.

## Discussion

The Lamiaceae family had the highest representation, with approximately 91 species spanning six subfamilies or tribes (**Figure 4**): Lamioideae, Mentheae, Ajugoideae, Ocimeae, Prostantheroideae, and Scutellarioideae. Ocimeae was the most represented sub-family, including 13 species of *Coleus*, 6 *Ocimum*, 4 *Plectranthus*, 3 *Eriope*, 2 *Aeollanthus*, and one species each of *Asterohyptis*, *Cantinoa*, and *Isodon*. Mentheae ranked second but contained the greatest species diversity within individual genera, represented by 14 species of *Salvia*, 4 *Nepeta*, 3 *Thymus*, 3 *Mentha*, and one species each of *Agastache*, *Origanum*, *Prunella*, *Satureja*, and *Dracocephalum*. The Scutellarioideae tribe was represented by 7 species of *Scutellaria* and 1 *Tinnea* species, Lamioideae by 2 species of *Stachys*, 3 *Lamium*, and one species each of *Marrubium*, *Leonotis*, and *Pseudodictamnus*. Ajugoideae comprised 10 *Teucrium* and 1 *Ajuga* species, whereas Prostantheroideae was represented solely by one species of *Prostanthera*.

Within the Lamiaceae family, several genera exhibited moderate to potent activity against *S. aureus* but showed no activity against the other two microbes. Active genera included *Coleus*, *Plectranthus*, *Aeollanthus*, *Salvia*, and *Tinnea*, which belong to the Ocimeae, Mentheae, and Scutellarioideae subfamilies.tribes. Plants from the Ocimeae and Mentheae groups are rich in diterpenoids, although of different types: Ocimeae species primarily produce abietanes and isopimaranes (Bitchagno et al., 2025c; Grayer et al., 2021), whereas Mentheae species additionally synthesize labdanes and clerodanes (Jassbi et al., 2016; Bonito et al., 2011). The two species studied from Scutellarioideae were dominated by flavonoids with a non-substituted B-ring and an additional oxidation on ring A, including compounds such as baicalin, baicalein, and dihydrobaicalin. In contrast, genera such as *Ocimum*, *Teucrium*, *Eriope*, *Lamium*, *Stachys*, and *Mentha*, for which multiple species were tested, showed no detectable activity against any of the pathogens. Some of these inactive genera, notably *Ocimum* and *Mentha*, are known to produce neither diterpenoids in potent amounts nor the characteristic Scutellarioideae-type flavonoids.

The Moraceae plants comprised *Dorstenia* (12 species) from Dorstenieae, *Ficus* (8 species) from Ficeae, *Maclura* (1 species) from Maclureae, *Morus* (1 species) from Moreae and *Artocarpus* (1 species) from Artocarpeae tribes. Both *Dorstenia* and *Ficus* are known to contain furanocoumarins, the active compounds proposed to induce the activity of *Dorstenia* species against *S. aureus*. Again, the concentration and type of these chemicals differ per species and might explain the differential profile of activity observed across these genera. The chemical profile found in *Maclura* is quite unique within the family as neither *Dorstenia* or *Ficus* are known to produce prenylated flavonoids. The furanocoumarins found in *Dorstenia* may also bear prenyl groups but prenylated flavonoids are rare. Similarly, no furanocoumarins were found in the fruits of *Maclura* examined during the study.

The four genera of Myrtaceae belong to the same subfamily, Myrtoideae, but of different tribes. There were 6 species of *Eucalyptus* from Eucalypteae, 1 species of *Kunzea* from Leptospermeae, 1 species of *Leptospermum* from Leptospermeae and 2 species of *Melaleuca* from Melaleuceae tribes. Except *Melaleuca* which has not been well studied, the three other genera are known as acylphloroglucinol producers, though they differ in their constitution. Most of the meroterpenes found in *Kunzea* and *Leptospermum* spp. are hybrid of flavonoids and terpenes. They do not produce formylphloroglucinols only found in *Eucalyptus* and identified as the main active components in the genus.

The diversity in the Fabaceae was seen within the Millettioids tribe as it was composed of *Pueraria* (1 species), *Pongamia* (1 species), *Lupinus* (1 species) and *Lonchocarpus* (1 species). The other three tribes of Faboideae used during the study included Phaseoleae with *Erythrina* (1 species), Leptolobieae with *Chaminodianthus* (3 species) and Sophoreae with *Sophora* (5 species). The only species of the family that belongs to a different subfamily, Caesalpinioideae, was *Peltophorum* (1 species) from the Peltophorum clade. Outside alkaloid fraction which showed activity from *Erythrina*, only *Lupinus* extracts proved active against *S. aureus* across the family. Like *Maclura*, *Lupinus* is known to produced prenylated isoflavones whose representatives of the plant are isoderrone, lupinisols A and B, lupinisoflavone L and wighteo (Hellal et al., 2021). Prenylated flavonoids were the actives identified from *Maclura* fruit extracts and fractions. Most of the Millettioid genera like *Pongamia* and *Lonchocarpus* are also known to produce rotenoids, a group of compounds considered as advanced isoflavonoids (Nchiozem-Ngnitedem et al., 2025). This group of compounds was not associated with antibacterial activity in this study.

On the other hand, differences in antibacterial activity within the same genus were primarily linked to variations in the levels of active metabolites among species. For instance, species of *Coleus* and *Plectranthus* were grouped into four clusters based on their abietane-type composition, with active coleons serving as the key distinguishing factors for one cluster, which was found in only a few species (Bitchagno et al., 2026). Additionally, while some species were enriched in abietane diterpenes, others predominantly accumulated flavonoids, a class of compounds generally not associated with antibacterial activity unless prenylated. In *Eucalyptus*, species also differed in their euglobal content, which was either very low, as observed in *E. perinnema*, or completely absent, as in *E. gunnii*. In these cases, the hexane extracts that displayed the strongest activity derived their potency from other metabolites. Active fractions from *E. perinnema* showed to be rich in triterpenes such as oleanolic and betulinic acids, whereas *E. gunnii* active fractions contained triglycerides and alkylphenols. Similarly, quinone levels varied among *Drosera* species, with *D. binata* and *D. macrantha subsp. macrantha* exhibiting the highest concentrations of plumbagin and droserone, correlating directly with their strongest activity against *S. aureus*.

Although a trend of activity was observed for *Daphne* extracts, further profiling could not be pursued due to insufficient plant material, as these are slow-growing species. In contrast, for genera in which multiple species were screened, antibacterial activity was either limited within the genus, as observed in *Hypericum*, or simply absent, as seen in *Phyllostachys*, *Withania*, *Impatiens*, *Anthurium*, *Teucrium*, *Mentha*, and *Lamium*.

All in all, this study highlights several classes of plant-derived chemicals as promising candidates for inhibiting *S. aureus*. The likelihood of finding potent anti-*S. aureus* compounds in plants is relatively high, as the chemical groups identified as drivers occur across multiple genera of various families of plants, such as prenylated flavonoids present of *Maclura*, and *Paulownia*. However, when considering these compounds as raw materials for industrial applications, the yield of active metabolites from different plant sources is of a critical importance and must be carefully evaluated to identify the most suitable candidates. Furthermore, relatively few extracts demonstrated activity against *E. coli*, despite numerous reports in the literature claiming the activity of plant-derived extracts and compounds against Gram-negative bacteria. For some extracts not active at a preliminary stage, the potency against *E. coli* becomes evident after fractioning the 60% MeOH extract. This might suggest that polar components are potential candidates to present a better prospect as it relates to *E. coli* inhibition. However, the pattern needs to be tested and validated by increasing the number of fractions from polar extracts to screen. Fractionation increases the concentration of active compounds into specific polar fractions.

Perhaps the most striking finding relates to the remarkable resistance of *A. brasiliensis* to all 462 plant preparations tested. As surprising as it might seem, this result challenges numerous previous reports as plant extracts and compounds are thought potently active against bacteria and fungi (Zhang et al., 2023). This raises intriguing questions, especially about the mechanisms by which pathogenic fungi resist antibiotics. The resistance observed may also underscore the need to explore alternative sources of naturals in which fungi represent a viable lead given the immense diversity of undescribed and uncharacterized fungal species.

## Data Availability

The data generated and/or analysed during the current study are available from the corresponding author (Gabin Bitchagno) on request.

## Supplementary material

Supplementary Table 1 S3 Detailed MIC values of samples tested.
