# Supplementary material for: Mapping antimicrobials in plant lineages
Source: Front Plant Sci. 2026 Sep 3;17:1936509. doi: 10.3389/fpls.2026.1936509 (PMC13582457; doi:10.3389/fpls.2026.1936509)
Supplement: Supplementary file 1 [file SupplementaryFile1.pdf]

Supplementary materials

Mapping Antimicrobials in Plant Lineages

Gabin Thierry M. Bitchagno,[1] * Paula Coates,[3] Erin Garcia,[2] Sohini Bhatia,[2] Amorita Correia,[1] Debbie Mulligan,[2] Scott Bintrim,[2] Monique S.J. Simmonds[1] *

[1]Royal Botanic Gardens, Kew, Richmond, London, TW9 3AE

[2]The Procter & Gamble Company, Mason Business Center, Mason, Ohio, USA

[3]The Procter & Gamble Company, 452 Basingstoke Rd, Reading, RG2 0RX, UK

646509

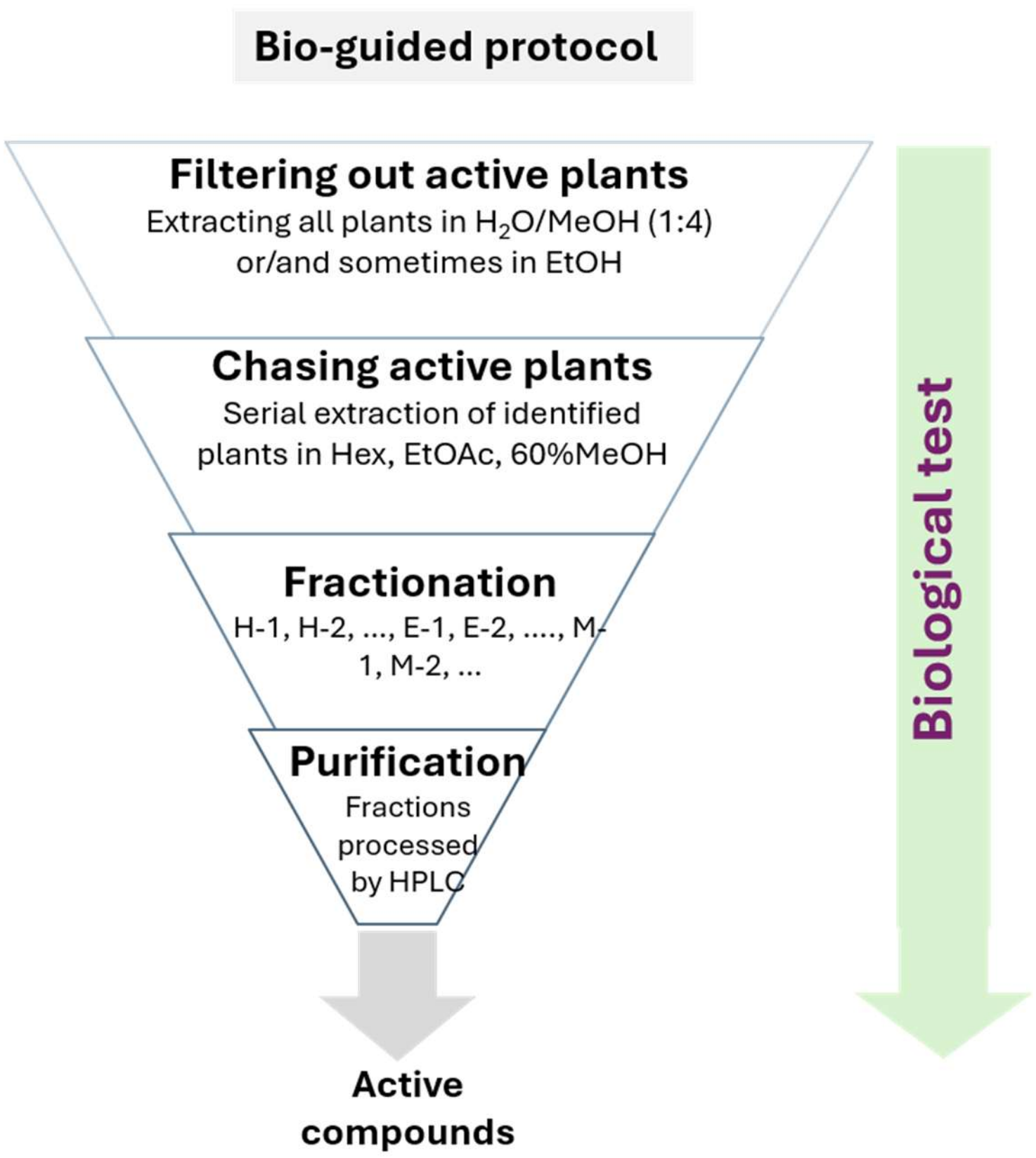

Figure S1. Schematic summary of the bio-guided protocol at the center of the study methodology

Table S1. List of plants used throughout the study

BI Number	Accession Number	Plant names as in the gardens	Accepted Latin binomial name	Plant organs	Date of collection
34461	1975-1274	*Abutilon mollisimum var. sandwicense*	*Abutilon grandifolium* (Willd.) Sweet	Aerial parts	09.08.2024
25386	1994-2792	*Acanthus spinosus*	*Acanthus spinosus* L.	Leaf, flower	18.08.2015
31519	1978-2809	*Achillea millefolium*	*Achillea millefolium* L.	Extract, Aerial parts	01.10.2013
23938	10083.01	*Actinidia chinensis*	*Actinidia chinensis* Planch.	Fruit water	19.11.2013
33230	1976-2374	*Aeollanthus buchniorianus*	*Aeollanthus buchnerianus* Briq.	Aerial parts	20.06.2023
33231	1970-4302	*Aeollanthus densiflorus*	*Aeollanthus densiflorus* Ryding	Aerial parts	20.06. 2023
23736	SN:PE116801-130902	*Agastache rugosa*	*Agastache rugosa* (Fisch. & C.A.Mey.) Kuntze	Extract	4.10.2013
21886	1993-99	*Agastache rupestris*	*Agastache rupestris* (Greene) Standl.	Leaves	10.7.2021
21884	2003-1986	*Ajuga reptans 'Braunherz'*	*Ajuga reptans* L.	Leaves	10.7.2012
31498	1995-2546	*Alpinia galangal*	*Alpinia galanga* (L.) Willd.	Whole plant	18.5.2021
31499	1986-2906	*Annona muricata*	*Annona muricata* L.	Aerial parts	18.05.2021
32507	1973-2200	*Anthurium gracile*	*Anthurium gracile* (Rudge) Lindl.	Fruit	13.10.2022
2060	1992-1973	*Anthurium huixtlense*	*Anthurium huixtlense* Matuda	Aerial roots	5.1.1995
32514	1984-8010	*Anthurium scandens*	*Anthurium scandens subsp. pusillum* Sheffer	Fruit	13.10.2022
32518	1968-38216	*Anthurium willdenowii*	*Anthurium willdenowii* Kunth	Fruit	13.10.2022
34486	1979-3512	*Aphelandra deppeana*	*Aphelandra scabra* (Vahl) Sm.	Leaves	25.02.1995

34481	1971-2768	*Apium graveolens*	*Apium sellowianum* H.Wolff	Branches and Leaves	21.08.2024
21961	1969-16839	*Arbutus unedo*	*Arbutus unedo* L.	Leaves	17.07.2012
31566	1987-81412	*Artocarpus heterophyllus*	*Artocarpus heterophyllus* Lam.	Leaves	21.07.2021
1643	1976-1283	*Asterohyptis stellulata*	*Asterohyptis stellulata* (Benth.) Epling	Aerial parts	28.07.1994
25397	1994-2788	*Astrantia major*	*Astrantia major* L.	Leaves	18.08.2015
33723<br>21875	2019-657<br>1957-48301	*Ballota pseudodictoamnus*	*Pseudodictamnus mediterraneus* Salmaki & Siadati	Aerial parts	1.11.2023
24181	SN: PD20601-131202	*Bambusa vulgaris*	*Bambusa balcooa* Roxb.	Leaf extract	11.02.2014
32200	1973-1861	*Banisteriopsis caapi*	*Banisteriopsis caapi* (Spruce ex Griseb.) C.V.Morton	Aerial parts	06.06.2022
1644	1992-1781	*Becium frutescens*	*Ocimum viphyense* A.J.Paton	Aerial parts	28.07.1994
1645	1992-1962	*Becium grandiflora*	*Ocimum grandiflorum* Lam.	Aerial parts	28.07.1994
31178	1997-5247	*Berberis pruinosa 'Punkata'*	*Berberis pruinosa* Franch.	Fruit	10.10.2021
21973	2008-705	*Berteroa ascendens*	*Berteroa mutabilis* (Vent.) DC.	Leaves	17.7.2012
21937	1982-344	*Betula aetnensis*	*Betula pendula subsp. pPendula*	Leaves	17.07.2012
21994	1973-10582	*Betula pendula*	*Betula pendula* Roth	Leaves	17.07.2012
23729	Batch no. 020.913	*Bletilla striata*	*Bletilla striata* (Thunb.) Rchb.f.	Cells in liquid and powder	1.10.2013
34462	1987-800	*Boesenbergia pandurata*	*Boesenbergia rotunda* (L.) Mansf.	Aerial parts	09.08.2024
21882	1970-1179	*Bryonia dioica*	*Bryonia alba* L.	Aerial parts	10.07.2012
21146	PMD280911	*Bupleurum chinense*	*Bupleurum chinense var. chinense*	Roots	28.9.2011
31850	1953-24719	*Camellia japonica*	*Camellia japonica* L.	Leaves, flower bud, twig	14.02.2022
33916	1975-4841	*Cantinoa mutabilis*	*Cantinoa mutabilis* (Rich.) Harley & J.F.B.Pastore	Aerial parts	20.11.2023
22728	1991-1826	*Castanea sativa*	*Castanea sativa* Mill.	Leaves and male flower	05.07.2012
31806	Not known	*Catharanthus roseus*	*Catharanthus roseus* (L.) G.Don	Leaves	2.2.2022
23850	1969-18283	*Centaurea aff nigra*	*Centaurea nigra* L.	Leaves	01.11.2013
31642	1969-10600	*Chamaecyparis pisifera filifera*	*Chamaecyparis pisifera* (Siebold & Zucc.) Endl.	Leaves and Twigs	21.09.2021
22703	1944-510	*Chusquea aff culeou*	*Chusquea culeou* É.Desv.	Aerial parts	12.3.2013
1829	1970-4296	*Coleus kilimandschariens*	*Coleus sp*	Leaves	14.09.1994
21934<br>30498	1965-10702<br>1965-10702	*Collinsonia canadensis*	*Collinsonia canadensis* L.	Leaves	17.07.2012<br>04.09.2020
31527	2004-2900	*Commiphora dulcis*	*Commiphora dulcis* Engl.	Bark	14.5.2021
33901	Not known	*Cornus sanguinea*	*Cornus sanguinea* L.	Stems and Branches	2024
22048	1976-4072	*Crambe maritima*	*Crambe maritima* L.	Leaves	19.07.2012
34464	1973-4418	*Crassula brevifolia ssp. Brevifolia*	*Crassula brevifolia subsp. bBrevifolia*	Aerial parts	09.08.2024
31658<br>31657	1971-4049<br>1971-4049	*Crataegus prunifolia*	*Crataegus persimilis* Sarg.	Leaves and Fruits	30-09-2021<br>30-09-2021
2055	1992-2044	*Croton trinitatis*	*Croton trinitatis* Millsp.	Leaves and flowers	04.01.1995
34299	1966-8207	*Daphne alpina*	*Daphne alpina* L.	Leaves	16.05.2024
33643<br>33646	2020-44	*Daphne bholua 'Jacqueline Postill'*	*Daphne bholua* Buch.-Ham. ex D.Don	Aerial parts	26.03.2024
34300	2016-108	*Daphne gemmata*	*Daphne gemmata* E.Pritz.	Leaves	26.03.2024
33647<br>33648<br>33927	2021-1056	*Daphne laureola*	*Daphne laureola* L.	Aerial parts	26.03.2024
34125	1977-4211	*Daphne pontica*	*Daphne pontica* L.	Leaves and Branches	26.03.2024
34126<br>34127	2013-29	*Daphne tangutica*	*Daphne tangutica* Maxim.	Leaves and Branches	26.3.2024
34341	1961-68701	*Dorstenia arifolia*	*Dorstenia arifolia* Lam.	Aerial parts	09.07.2024
34342	1977-2803	*Dorstenia bahiensis*	*Dorstenia bahiensis* Klotzsch ex Fisch. & C.A.Mey.	Aerial parts	9.7.2024
31855	1990-2304	*Dorstenia barteri*	*Dorstenia barteri* Bureau	Leaves and Twigs	25.02.2022
34337	1990-2304	*Dorstenia barteri var. multiradiata*	*Dorstenia barteri var. multiradiata* (Engl.) Hijman & C.C.Berg	Aerial parts	09.07.2024
34339	1979-3484	*Dorstenia brownii*	*Dorstenia brownii* Rendle	Aerial parts	09.07.2024
34333	2012-1414	*Dorstenia christenhuszii*	*Dorstenia christenhuszii* M.W.Chase & M.F.Fay	Aerial parts	9.7.2024
34340	2000-4318	*Dorstenia contrajerva*	*Dorstenia contrajerva* L.	Aerial parts	9.7.2024
34334	1970-3608	*Dorstenia foetida*	*Dorstenia foetida* (Forssk.) Schweinf.	Aerial parts	09.07.2024
34345	1990-1303	*Dorstenia foetida subs. Lancifolia*	*Dorstenia foetida subsp. lancifolia* (Rendle) Friis	Aerial parts	09.07.2024
34336	1967-35803	*Dorstenia gigas*	*Dorstenia gigas* Schweinf. ex Balf.f.	Aerial parts	9.7.2024
34338	2022-590	*Dorstenia hildebrandtii*	*Dorstenia hildebrandtii* Engl.	Aerial parts	9.7.2024
34343	1969-17745	*Dorstenia psilirus*	*Dorstenia psilurus* Welw.	Aerial parts	9.7.2024
21902	2009-3042	*Dracocephalum stamineum*	*Dracocephalum stamineum* Kar. & Kir.	Leaves	10.7.2012
33845<br>33846	1969-23754<br>1969-23754	*Dracontomelon dao*	*Dracontomelon dao* (Blanco) Merr. & Rolfe	Leaves and Branches	23.01.2024<br>23.01.2024
34459	1998-1463	*Drosera affinis*	*Drosera affinis* Welw. ex Oliv.	Aerial parts	Before 2000
34639	2014-2354	*Drosera aliciae*	*Drosera aliciae* Raym.-Hamet	Leaves and flowers	6.2.2025
34573	1986-646	*Drosera binata*	*Drosera binate* Labill.	Leaves and flowers	6.2.2025
34636	2002-1329	*Drosera capensis*	*Drosera capensis* L.	Leaves and flowers	6.2.2025
34575	2001-2740	*Drosera dichrosepala*	*Drosera dichrosepala* Turcz.	Leaves and flowers	6.2.2025
34569	2019-2556	*Drosera gibsonii*	*Drosera gibsonii* P. Mann	Leaves and flowers	6.2.2025
34640	2008-2086	*Drosera macrantha subsp. Macrantha*	*Drosera macrantha subsp. Macrantha*	Leaves and flowers	6.2.2025
34463	1989-00233	*Drosera platystigma*	*Drosera platystigma* Lehm.	Aerial parts	09.08.2024
34635	2009-3256	*Drosera venusta*	*Drosera venusta* Debbert	Leaves and flowers	6.2.2025
1647	1992-2261	*Eriope filifolia*	*Eriope filifolia* Benth.	Aerial parts	28.7.1994
1648	1971-3802	*Eriope hypenioides*	*Eriope hypenioides* Mart. ex Benth.	Aerial parts	28.07.1994
33232	1977-1362	*Eriope Macrostachys*	*Eriope macrostachya* Mart. ex Benth.	Aerial parts	20.06.2023
31841<br>31842<br>32071	2017-908	*Erythrina lysistemon*	*Erythrina lysistemon* Hutch.	Leaves, Twigs and Bark	14.1.2022
30980	1972-6023	*Eucalyptus aggregata*	*Eucalyptus aggregata* H.Deane & Maiden	Seed	21\10\2020
30986	1988-8117	*Eucalyptus chapmaniana*	*Eucalyptus chapmaniana* Cameron	Fruit	03.11.2020
33370	1972-6025	*Eucalyptus dalrympleana*	*Eucalyptus dalrympleana* Maiden	Fruit	04.09.2023
30987<br>33564	2003-2184<br>1998-164	*Eucalyptus gunnii*	*Eucalyptus gunnii* Hook.f.	Fruit	3.11.2020
31852	1977-6267	*Eucalyptus pauciflora*	*Eucalyptus pauciflora* Sieber ex Spreng.	Fruit	21.02.2022
30376	1972-6027	*Eucalyptus perriniana*	*Eucalyptus perriniana* F.Muell. ex Rodway	Fruit	20.08.2020

30985	1972-4176				03.11.2020
19484	911-30-91101	*Eucommia ulmoides*	*Eucommia ulmoides* Oliv.	Flower	22.4.2010
33708	External provider	*Euphorbia pulcherima*	*Euphorbia pulcherrima* Willd. ex Klotzsch	Whole plant	4.12.2023
25305	1984-4518	*Ficus benghalensis*	*Ficus benghalensis* L.	Leaves and Twigs	16.06.2015
18617	1973-15025	*Ficus carica*	*Ficus carica* L.	Fruit	09.06.2009
31565<br>31674	1964-28021<br>1964-2802	*Ficus cordata*	*Ficus cordata* Thunb.	Leaves	21.07.2021<br>11.10.2021
31674	1964-2802	*Ficus coronata*	*Ficus coronata* Spin	Leaves	11.10.2021
34494	Not known	*Ficus lingua*	*Ficus lingua* Warb. ex De Wild. & T.Durand	Aerial parts	Before 2000
34472<br>34485	1969-13673<br>1969-13673	*Ficus lyrata*	*Ficus lyrata* Warb.	Branches and Leaves	20.08.2024<br>20.10.1999
31574	1984-4525	*Ficus religiosa*	*Ficus religiosa* L.	Stems	12.08.2021
31501	1986-3428	*Ficus thonningii*	*Ficus thonningii* Blume	Aerial parts	18.05.2021
21883<br>22171<br>22172	2003-1181<br>1969-50309	*Filipendula ulmaria*	*Filipendula ulmaria* (L.) Maxim.	Aerial parts, fruit	10.7.2012
22174<br>23495	1978-570	*Filipendula vulgaris*	*Filipendula vulgaris* Moench	Leaves, Fruit and Twigs	17.08.2012
23735	SN:PE116701-13902	*Forsythia koreana*	*Forsythia koreana* (Rehder) Nakai	Extract	4.10.2013
31564	External provider	*Galium aparine*	*Galium aparine* L.	Seeds	21.07.2021
21974	1955-3704	*Galium purpureum*	*Thliphthisa purpurea* (L.) P.Caputo & Del Guacchio	Leaves	17.07.2012
21918	Not known	*Galium verum*	*Galium verum* L.	Leaves and Flowers	13.07.2012
33717	2017-1905	*Garcinia livingstonei*	*Garcinia livingstonei* T. Anderson	Aerial parts	1.11.2023
23727	Batch no. 260.813	*Gardenia jasminoides*	*Gardenia jasminoides* J.Ellis	Cells in liquid, Cells in powder and extract	1.10.2013
31627	1970-4012	*Geranium kotschyi charlesii*	*Geranium kotschyi* Boiss.	Roots	01.09.2021
25381	Not known	*Geranium phaeum*	*Geranium phaeum* L.	Aerial parts	18.08.2015
34477	1989-2129	*Geum coccinuem*	*Geum coccineum* Sm.	Leaves and Branches	27.08.2024
23848<br>23940	1969-13271<br>0761476	*Ginkgo biloba*	*Ginkgo biloba* L.	Leaves and Extract	31.10.2013<br>19.11.2013
33902	2010-1059	*Hedychium coccineum*	*Hedychium coccineum* Buch.-Ham. ex Sm.	Aerial parts	18.3.2024
22200	2010-1448	*Helianthemum nummularium*	*Helianthemum cinereum subsp. cCinereum*	Leaves	22.8.2012
31625	1980-1719	*Helichrysum arwae*	*Helichrysum arwae* J.R.I.Wood	Aerial parts and twigs	01.09.2021
27820	Sir Harold Hillier Gardens: 6	*Hypericum addingtonii*	*Hypericum addingtonii* N.Robson	Leaves and Flowers	7.8.2017
32008<br>30989	1988-4836<br>2012-532	*Hypericum forrestii*	*Hypericum forrestii* (Chitt.) N.Robson	Aerial parts and Fruits	29.04.2022
27779	Sir Harold Hillier Gardens: 50	*Hypericum henryi (ssp. henryi)*	*Hypericum henryi subsp. hHenryi*	Leaves and Flowers	7.8.2017
27857	Sir Harold Hillier Gardens: 56	*Hypericum hircinium*	*Hypericum hircinum* L.	Leaves and fruits	7.8.2017
27782	Sir Harold Hillier Gardens: 113	*Hypericum hookerianum*	*Hypericum hookerianum* Wight & Arn.	Leaves and Flowers	7.8.2017
27764	Sir Harold Hillier Gardens: 35	*Hypericum latisepalum*	*Hypericum latisepalum* (N.Robson) N.Robson	Leaves and Flowers	7.8.2017
27831	Sir Harold Hillier Gardens: 117	*Hypericum stellatum*	*Hypericum stellatum* N.Robson	Leaves and Flowers	7.8.2017
33899<br>33900	2005-2481	*Impatiens balansae*	*Impatiens balansae* Hook.f.	Aerial parts	13.3.2024
34460	1945-22601	*Impatiens niamniamensis*	*Impatiens niamniamensis* Gilg	Aerial parts	09.08.2024
21917	Not known	*Impatiens parviflora*	*Impatiens parviflora* DC.	Aerial parts	13.7.2012
34456	1952-36503	*Justicia nyassana*	*Justicia nyassana* Lindau	Aerial parts	09.08.2024
34479	1993-1871	*Kunzea ericoides*	*Kunzea ericoides* (A.Rich.) Joy Thomps.	Leaves and Branches	21.8.2024
21968	1940-9205	*Lamium flexuosum*	*Lamium flexuosum* Ten.	Leaves	17.07.2012
21971	1966-5603	*Lamium galeobdolon*	*Lamium galeobdolon* (L.) L.	Leaves	17.07.2012
21975	1969-19284	*Lamium maculatum*	*Lamium maculatum* (L.) L.	Leaves	17.07.2012
34483	1969-13987	*Leea horrida*	*Leea angulata* Korth. ex Miq.	Roots	8.09.1994
1817	1991-1166	*Leonotis ocymifolia Vaimsriana*	*Leonotis ocymifolia* (Burm.f.) Iwarsson	Leaves	14.09.1994
34476	1990-2979	*Leptospermum continentale*	*Leptospermum continentale* Joy Thomps.	Leaves and Branches	21.08.2024
34475	1988-1037	*Leptospermum lanigerum*	*Leptospermum lanigerum* (Aiton) Sm.	Leaves and Branches	21.08.2024
29098<br>30765<br>33888	1984-5377	*Lonchocarpus costaricensis*	*Lonchocarpus costaricensis* (Donn.Sm.) Pittier	Leaves, Twigs and Seeds	2020s
33641<br>33642	2020-167	*Lupinus arboreus*	*Lupinus arboreus* Sims	Aerial parts	5.10.2023
31452<br>31757	1910-66603<br>1910-66601	*Maclura pomifera*	*Maclura pomifera* (Raf.) C.K.Schneid.	Fruit	14.052021<br>02.11.2021
33368	2001-2001	*Mahonia lomariifolia*	*Berberis oiwakensis* (Hayata) Laferr.	Leaves and Twigs	31.08.2023
21879	1977-1041	*Marrubium cylleneum Boiss & Heldr*	*Marrubium cylleneum* Boiss & Heldr	Aerial parts	10.07.2012
34478	1982-1371	*Melaeuca rhaphiophylla*	*Melaleuca rhaphiophylla* Schauer	Leaves and Branches	20.06.2023
34474	1987-3615	*Melaleuca Citrina*	*Melaleuca citrina* (Curtis) Dum.Cours.	Leaves and Branches	21.08.2024
21885	1978-852	*Mentha longifolia*	*Mentha longifolia* (L.) L.	Leaves	10.07.2012
21970	1972-6406	*Mentha spicata*	*Mentha spicata* L.	Aerial parts	17.07.2012
21969	1969-9293	*Mentha villosa*	*Mentha × villosa* Huds.	Leaves	17.07.2012
22005	1972-82	*Mimulus cardinalis*	*Erythranthe cardinalis* (Douglas ex Benth.) Spach	Aerial parts	17.07.2012
23934	0721184	*Morus alba*	*Morus alba* L.	Leaves	19.11.2013
21901	1991-294	*Nepeta faassenii*	*Nepeta × faassenii* Bergmans ex Stearn	Aerial parts	10.7.2012
21896	1998-3053	*Nepeta menthoides*	*Nepeta menthoides* Boiss. & Buhse	Aerial parts	10.7.2012
22039	1983-3408	*Nepeta sibirica*	*Nepeta sibirica* L.	Aerial parts	19.07.2012
31502	1944-13201	*Newbouldia laevis*	*Newbouldia laevis* (P.Beauv.) Seem. ex Bureau	Aerial parts	18.05.2021
32558	Waitrose (best before: 13 Nov)	*Ocimum basilicum (var. thyrsiflora)*	*Ocimum basilicum var. basilicum*	Leaves	10.11.2022
33130	1993-1030	*Ocimum gratissimum*	*Ocimum gratissimum* L.	Aerial parts	1990s

4412	1996-916	*Ocimum kilimandscharicium*	*Ocimum kilimandscharicum* Gürke	Flowers	1.10.1996
4319	1995-3474	*Ocimum minimum*	*Ocimum basilicum var. minimum* (L.) Alef.	Aerial parts	4.9.1996
2311	1992-1965	*Odontonema tubiforne*	*Odontonema tubaeforme* (Bertol.) Kuntze	Leaves	24.04.1995
2043	1991-1775	*Omphalea hypolenca*	*Omphalea hypoleuca* Griseb.	Leaves	04.01.1995
23377<br>23926<br>23927	1969-16374<br>1969-16375	*Ophiopogon japonicus*	*Ophiopogon japonicus* (Thunb.) Ker Gawl.	Leaves, Fruits and Roots	13.11.2013
21892<br>21960<br>31516	1995-77<br>1973-18222<br>1969-19318	*Origanum Vulgare*	*Origanum vulgare* L.	Aerial parts	10.7.2012
21657	EBC 83755	*Paeonia lactiflora*	*Paeonia lactiflora* Pall.	Roots	24.4.2012
21658	EBC 83756	*Paeonia ostii*	*Paeonia ostii* T.Hong & J.X.Zhang	Roots	24.4.2012
31997<br>31998<br>33565	2002-723<br>2001-2068	*Paulownia fargesii*	*Paulownia fargesii* Franch.	Fruits and Twigs	14.4.2022
31995	2000-4858	*Paulownia tomentosa*	*Paulownia tomentosa* (Thunb.) Steud.	Fruits	14.4.2022
32531	2004-2563	*Pelargonium crispum 'Variegatum'*	*Pelargonium crispum* (P.J.Bergius) L'Hér.	Leaves	19.102022
33847<br>33849	2012-154<br>93595	*Peltophorum dubium*	*Peltophorum dubium* (Spreng.) Taub.	Leaves and Branches	23.1.2024
34480	1972-6329	*Persicaria capitata*	*Persicaria capitata* (Buch.-Ham. ex D.Don) H.Gross	Leaves	21.08.2024
23726	NA22187 (Lot #C2047)	*Phoenix dactylifera*	*Phoenix dactylifera* L.	Fruit extract	1.10.2013
33707	2009-242	*Phyllanthus casticum*	*Phyllanthus casticum* P.Willemet	Leaves	12.3.2013
23935	AHXT2	*Phyllanthus embilica*	*Phyllanthus emblica* L.	Fruit	19.11.2013
29283	1984-2279	*Phyllostachys bambusoides*	*Phyllostachys reticulata* (Rupr.) K.Koch	Leaves, Shoot bark, and Stems	29.03.2019
22766	1992-1625	*Phyllostachys bambusoides "Castillonis"*	*Phyllostachys reticulata* (Rupr.) K.Koch	Leaf, cane and stem	12.04.13
29494<br>22705<br>22706<br>22762<br>22763<br>22764<br>22765	1995-2198<br>1985-2847<br>1973-20183	*Phyllostachys nigra*	*Phyllostachys nigra* (Lodd. ex Lindl.) Munro	Aerial parts and Cane	12.3.2013
22701<br>22702	1998-3902	*Phyllostachys viridi-glaucescens*	*Phyllostachys viridiglaucescens* (Carrière) Rivière & C.Rivière	Aerial parts	12.3.2013
22709<br>22710<br>22713	1995-3996<br>1989-315	*Phyllostachys vivax*	*Phyllostachys vivax* McClure	Aerial parts	12.3.2013
32201	2010-1732	*Piper betel*	*Piper betle* L.	Leaves	8.6.2022
34484	1982-534	*Piper Nigrum*	*Piper nigrum* L.	Leaves	10.09.2024
33229	1976-1005	*Plecthrantus ciliatus*	*Plectranthus ciliatus* E.Mey.	Aerial parts	20.06.2023
33227	1959-79901	*Plecthrantus coleoides*	*Coleus paniculatus* Benth.	Aerial parts	20.06.2023
1671	1970-2059	*Plecthrantus crassus*	*Coleus crassus* (N.E.Br.) Culham	Aerial parts	28.07.1994
33220	1970-3770	*Plecthrantus cyaneus*	*Coleus hadiensis* (Forssk.) A.J.Paton	Aerial parts	20.06.2023
33222	ST100	*Plecthrantus ecklonii*	*Plectranthus ecklonii* Benth.	Aerial parts	1990s
33221	ST103	*Plecthrantus elegans*	*Plectranthus elegans* Britten	Aerial parts	1990s
33218	1990-1148	*Plecthrantus fruticosus*	*Coleus fruticosus* Wight ex Benth.	Aerial parts	20.06.2023
33224	1970-3783	*Plecthrantus grandis*	*Coleus barbatus var. grandis* (L.H.Cramer) A.J.Paton	Aerial parts	20.06. 2023
33225	1977-3287	*Plecthrantus hadiensis*	*Coleus hadiensis* (Forssk.) A.J.Paton	Aerial parts	20.06.2023
33226	1977-1638	*Plecthrantus hyemalis*	*Coleus hymalis* (J.R.I.Wood) A.J.Paton	Aerial parts	20.06.2023
33228	1980-2707	*Plectranthus cicatricosus*	*Coleus burorum* Chiov.	Aerial parts	20.06.2023
33219	1990-3233	*Plectranthus coerulens*	*Coleus coeruleus* Gürke	Aerial parts	20.06.2023
33223	ST133	*Plectranthus cylindraseus*	*Coleus cylindraceus* (Hochst. ex Benth.) A.J.Paton	Aerial parts	1990s
22199	1981-2225	*Polygala chamaebuxus var grandiflora*	*Chamaebuxus alpestris* Spach	Leaves	22.08.2012
23987	1988-5752	*Polygonatum odoratum*	*Polygonatum odoratum* (Mill.) Druce	Leafs, Roots, Rhizome and Tuber	13.11.2013
23743	2008-1917	*Pongamia pinnata*	*Pongamia pinnata* (L.) Pierre	Leaves	2013
34488	3595-0	*Portulaca oleracea*	*Portulaca oleracea* L.	Aerial parts	1990s
22034	1996-2676	*Prostanthera cuneata*	*Prostanthera cuneata* Benth.	Leaves	19.7.2012
25389	Not known	*Prunella vulgaris*	*Prunella vulgaris* L.	Leaves	18.8.2015
23319	Code: HDG050, Batch:GG20120801	*Pueraria lobata*	*Pueraria montana var. lobata* (Willd.)	Roots	15.7.2013
31524	1977-4237	*Punica granatum*	*Punica granatum* L.	Flowers, Fruit Shells, and Pericarp	22.06.2021
33774	1969-425<br>2009-96	*Punica granatum var. nana*	*Punica granatum* L.	Leaves	23.01.2024
29282	1989-315	*Qiongzhuea tumidiana*	*Chimonobambusa tumidissinoda* Ohrnb.	Leaves	29.03.2019
31987	1977-6792<br>1977-6792	*Quercus infectoria veneris*	*Quercus infectoria* G.Olivier	Leaves, twigs, fruit	22.04.2022
1694	1983-4703	*Rabdosia rugova*	*Isodon rugosus* (Wall. ex Benth.) Codd	Aerial parts	02.08.1994
22036	2003-1381	*Ribes speciosum*	*Ribes speciosum* Pursh	Leaves	19.7.2012
22198	1993-1758	*Rumex rupestris*	*Rumex rupestris* Le Gall	Leaves	22.8.2012
25394	Not known	*Ruscus aculeatus*	*Ruscus aculeatus* L.	Leaves	18.08.2015
31045	2016-2181	*Salvia involucrata 'Bethellii'*	*Salvia involucrata* Cav.	Aerial parts	2020
21899	2008-1458	*Salvia aethiopis*	*Salvia aethiopis* L.	Leaves	10.7.2012
21877	1972-10374	*Salvia blancoana (?)*	*Salvia blancoana* Webb & Heldr.	Leaves	10.07.2012
21880	1973-21577	*Salvia candelabrum*	*Salvia candelabrum* Boiss.	Aerial parts	10.07.12
33884	1995-3910	*Salvia divinorum*	*Salvia divinorum* Epling & Játiva	Aerial parts	4.3.2024
33887	1969-51866	*Salvia dorisiana*	*Salvia dorisiana* Standl.	Aerial parts	04.03.2024
33885	1994-2122	*Salvia elegans*	*Salvia elegans* Vahl	Aerial parts	4.3.2024
33886	1970-4915	*Salvia gesneriflora*	*Salvia gesneriflora* Lindl. & Paxton	Aerial parts	04.03.2024
21900	1986-6320	*Salvia glutinosa*	*Salvia glutinosa* L.	Leaves	10.07.2012
31044	2015-2258	*Salvia mexicana 'Limelight'*	*Salvia mexicana* L.	Aerial parts	2020
31525	2018-532	*Salvia officinalis*	*Salvia officinalis* L.	Aerial parts	2021
33369<br>21887	1978-4849<br>1986-6328	*Salvia sclarea*	*Salvia sclarea* L.	Leaves	31.8.2023
25393	Not known	*Salvia verticillata*	*Salvia verticillata* L.	Leaves	18.08.2015
21876	1938-9809	Salvia viridis 'Rosea'	Salvia viridis L.	Aerial parts and Flowers	10.07.2012
32486	1984-5404	*Sambucus adnata*	*Sambucus adnata* Wall. ex DC.	Aerial parts	12.10.2022

19954	399901 LOT 6	*Sambucus nigra 'Black Lace'*	*Sambucus nigra* L.	Flower Extract	27.8.2010
32516	1985-2822	*Sanseviera subspicata*	*Dracaena subspicata* (Baker) Byng & Christenh.	Fruit	13.10.2022
34467	1957-63005	*Sarcostemma viminale*	*Cynanchum viminale* (L.) L.	Aerial parts	09.08.2024
22707	1975-14422	*Sasa palmata*	*Sasa palmata* (Burb.) E.G.Camus	Aerial parts	12.03.2013
29190	1996-4675	*Sasa senanensis*	*Sasa senanensis* (Franch. & Sav.) Rehder	Leaves	2019
25392<br>25398	Not known	*Satureja montana*	*Satureja montana* L.	Leaves	18.8.2015
25384<br>25385	Not known	*Saxifraga spathularis*	*Saxifraga spathularis* Brot.	Leaves and Flowers	18.8.2015
32506	2010-673	*Scadoxus nutans*	*Scadoxus nutans* (Friis & I.Bjørnstad) Friis & Nordal	Fruit	13.10.2022
21881	1988-1442	*Scrophularia grandiflora*	*Scrophularia grandiflora* DC.	Aerial parts	10.07.2012
33134	Not known	*Scutellaria albida*	*Scutellaria albida* L.	Aerial parts	1.8.1994
33135	Not known	*Scutellaria amana*	*Scutellaria tomentosa* Bertol.	Aerial parts	1.8.1994
4446	1986-4720	*Scutellaria baicalensis*	*Scutellaria baicalensis* Georgi	Aerial parts	08.10.1996
33131	Not known	*Scutellaria barbata*	*Scutellaria barbata* D.Don	Aerial parts	1.8.1994
33132	Not known	*Scutellaria diffusa*	*Scutellaria diffusa* Benth.	Aerial parts	1.8.1994
21888<br>4435	1996-1696<br>1986-4833	*Scutellaria galericulata*	*Scutellaria galericulata* L.	Aerial parts	10.07.2012<br>03.10.1996
21874	1982-257	*Scutellaria tournefortii*	*Scutellaria tournefortii* Benth.	Aerial parts	10.07.2012
31849	1986-8316	*Sequoia sempervirens*	*Sequoia sempervirens* (D.Don) Endl.	Fruits	14.02.2022
34466	1996-354	*Silene caryophylloides subsp. echinus*	*Silene caryophylloides subsp. echinus* (Boiss. & Heldr.) Coode & Cullen	Aerial parts	2024
25390	2024-0319	*Silene coronaria*	*Silene coronaria* (L.) Clairv.	Leaves	18.8.2015
25391<br>25395	Not known	*Silene verticillata*	*Silene spergulifolia* (Willd.) M.Bieb.	Leaves and Flowers	18.08.2015
23734<br>23763	lot S139012C13	*Silybum marianum*	*Silybum marianum* (L.) Gaertn.	Extract and Powder extract	4.10.2013
31798<br>31652<br>31794<br>31653<br>31798	Orangery garden	*Solanum laciniatum*	*Solanum laciniatum* Aiton	Fruit, Roots, and Bark	13.12.2021
1712	1970-3550	*Solenostemon shirensis*	*Coleus shirensis* Gürke	Aerial parts	1990s
23937	D14527	*Sophora angustifolia*	*Sophora flavescens var. flavescens*	Roots	19.11.2013
23983<br>23984<br>23985<br>23986	EBC 82203	*Sophora flavescens*	*Sophora flavescens* Aiton	Roots	27.11.2013
33721	1997-6552	*Sophora fullida*	*Sophora fulvida* (Allan) Heenan & de Lange	Aerial parts	1.11.2023
33706	1995-3390	*Sophora microphylla*	*Sophora microphylla* Aiton	Aerial parts	1.11.2023
33711	1995-2245	*Sophora tetraptera*	*Sophora tetraptera* J.S.Muell.	Aerial parts	1.11.2023
23732	23732	*Sorbus aucuparia*	*Sorbus aucuparia* L.	Fruit extract	4.10.2013
32440<br>31668	1982-3<br>1982-3	*Sorbus takhtajanii*	*Hedlundia takhtajanii* (Gabrieljan) Mezhenskyj	Fruit	13.09.2022<br>24.09.2021
21890<br>21891<br>21897	2003-26<br>2004-2738<br>1998-626	*Stachys byzantina*	*Stachys byzantina* K.Koch	Leaves	10.7.2012
21889	1972-2684	*Stachys germanica*	*Stachys germanica* L.	Leaves	10.07.2012
22013	2003-3763	*Stachys macrantha*	*Betonica macrantha* K.Koch	Leaves	18.07.2012
21895	1969-1976	*Stachys officinalis*	*Betonica officinalis* L.	Leaves	10.07.2012
22771	H.C. de Lima et al 2776	*Staminodianthus duckei*	*Staminodianthus duckei* (Yakovlev)	Leaves	24.11.1986
22772	N.Y. Sandwith 1393	*Staminodianthus racemosus*	*Staminodianthus racemosus* (Hoehne) D.B.O.S.Cardoso & H.C.Lima	Leaves	6.9.1937
22773	C.A. Cid Ferreira et al. 7129	*Staminodianthus rosae*	*Staminodianthus rosae* (H.C.Lima) D.B.O.S.Cardoso & H.C.Lima	Leaves	22.4.1986
34487	Not known	*Sterculia mexicana*	*Sterculia mexicana* R.Br.	Roots	4.9.1995
33898	1969-50088	*Strobilanthes glutinosa*	*Strobilanthes ixiocephala* Benth.	Aerial parts	18.03.2024
23737<br>23742	Batch TES-CO-110401	*Terminalia sericea*	*Terminalia sericea* Burch. ex DC.	Extract and Powder	4.10.2013
22040	1993-422	*Teucrium asiaticum*	*Teucrium asiaticum* L.	Leaves	1990s
4130	1995-3340	*Teucrium betonicum*	*Teucrium betonicum* L'Hér.	Leaves	1990s
21959	1981-3202	*Teucrium chamaedrys*	*Teucrium chamaedrys* L.	Leaves	17.07.2012
1789	1981-5945	*Teucrium kotschyanum*	*Teucrium kotschyanum* Poech	Leaves	9.8.1994
22016	1986-887	*Teucrium marum*	*Teucrium marum* L.	Leaves	18.07.2012
1786	1974-1178	*Teucrium montanum*	*Teucrium montanum* L.	Leaves	9.8.1994
1714	1969-19375	*Teucrium orientale*	*Teucrium orientale* L.	Aerial parts	01.08.1994
33217	1969-16375	*Teucrium polium*	*Teucrium polium* L.	Aerial parts	1990s
31637	1981-4139	*Teucrium polium 'Polivan'*	*Teucrium polium* L.	Aerial parts	01.09.2021
33216	1970-2427	*Teucrium scorodonia*	*Teucrium scorodonia* L.	Aerial parts	20.06.2023
21878<br>22012<br>22015	1960-66706<br>1977-227<br>1960-66706	*Teucrium subspinosum*	*Teucrium subspinosum* Pourr. ex Willd.	Leaves	10.07.2012<br>18.07.2012<br>18.07.2012
1783	Not known	*Teucrium webbianium*	*Teucrium webbianum* Boiss.	Leaves	09.08.1994
21894	2004-2740	*Thymus herba-barona*	*Thymus herba-barona* Loisel.	Aerial parts	10.7.2012
21972	1969-19395	*Thymus serpyllum*	*Thymus serpyllum* L.	Leaves	17.07.2012
31624	1969-19304	*Thymus vulgaris*	*Thymus vulgaris* L.	Aerial Parts, Twigs and Flowers	01.09.2021
29880	1973-16687	*Tilia aff.rubra*	*Tilia platyphyllos subsp. cCorinthiaca*	Leaves	13.08.2019
29881	1903-4804	*Tilia euchlora*	*Tilia × euchlora* K.Koch	Leaves	13.08.2019
29879	2010-2417	*Tilia tomentosa'Brabant'*	*Tilia tomentosa* Moench	Leaves	13.8.2019
29882	1973-16684	*Tilia x europaea*	*Tilia × europaea* L.	Leaves	13.08.2019
3210	1956-46103	*Tinnea zambesiaca*	*Tinnea zambesiaca* Baker	Whole plant	12.10.1995
33727	1992-3564	*Turnera ulmunifolia*	*Turnera ulmifolia* L.	Aerial parts	01.11.2023
31518	1969-50660	*Valeriana officinalis*	*Valeriana officinalis* L.	Whole plant	11.06.2021
33733	1936-437075	*Vanda tricolor*	*Vanda tricolor* Lindl.	Whole plant	1.11.2023
34465	1958-3207	*Verbascum dumulosum*	*Verbascum dumulosum* P.H.Davis & Hub.-Mor.	Aerial parts	09.08.2024
31626	1975-3858	*Verbascum spinosum*	*Verbascum spinosum* L.	Aerial parts	01.09.2021
21916	2006-1419	*Verbascum x hybridium*	*Verbascum nigrum subsp. nNigrum*	Flowers	13.07.2012
31990	1986-2802	*Vernonia amygdalina*	*Gymnanthemum amygdalinum* (Delile) Sch.Bip.	Leaves	25.04.2022
31532	2015-842	*Withania aristata*	*Withania aristata* (Aiton) Pauquy	Roots	14.05.2021
31530	1999-993	*Withania begoniifolia*	*Mellissia begoniifolia* (Roxb.) Hook.f.	Roots	14.5.2021
31529	1964-53502	*Withania riebeckii*	*Withania riebeckii* Schweinf. ex Balf.f.	Roots	14.05.2021
34133	External provider	*Withania somnifera*	*Withania somnifera* (L.) Dunal	Aerial parts	16.05.2024
33718<br>33719	2013-1484	*Zanthoxylum heterophyllum*	*Zanthoxylum heterophyllum* (Lam.) Sm.	Aerial parts	1.11.2023

Table S2. MIC (µg/mL) values of preliminary extracts

	SA	EC
	MIC	MIC
Ginkgoales Ginkgoaceae Ginkgo biloba	>1000	>1000
Pinales Cupressaceae Sequoia sempervirens	62.5	>1000
Pinales Cupressaceae Chamaecyparis pisifera	>1000	>1000
Magnoliales Annonaceae Annona muricata	>1000	>1000
Piperales Piperaceae Piper betle	>1000	>1000
Piperales Piperaceae Piper nigrum	>1000	>1000
Alismatales Araceae Anthurium scandens subsp pusillum	>1000	>1000
Alismatales Araceae Anthurium huixtlense	>1000	>1000
Alismatales Araceae Anthurium gracile	>1000	>1000
Alismatales Araceae Anthurium willdenowii	>1000	>1000
Asparagales Orchidaceae Bletilla striata	>1000	>1000
Asparagales Orchidaceae Vanda tricolor	>1000	>1000
Asparagales Amaryllidaceae Scadoxus nutans	>1000	>1000
Asparagales Asparagaceae Dracaena subspicata	>1000	>1000
Asparagales Asparagaceae Ruscus aculeatus	>1000	>1000
Asparagales Asparagaceae Polygonatum odoratum	>1000	>1000
Asparagales Asparagaceae Ophiopogon japonicus	>1000	>1000
Arecales Arecaceae Phoenix dactylifera	>1000	>1000
Zingiberales Zingiberaceae Hedychium coccineum	>1000	>1000
Zingiberales Zingiberaceae Boesenbergia rotunda	>1000	>1000
Zingiberales Zingiberaceae Alpinia galanga	>1000	>1000
Poales Poaceae Chusquea culeou	>1000	>1000
Poales Poaceae Bambusa balcooa	>1000	>1000
Poales Poaceae Sasa palmata	>1000	>1000
Poales Poaceae Sasa senanensis	>1000	>1000
Poales Poaceae Chimonobambusa tumidissinoda	>1000	>1000
Poales Poaceae Phyllostachys reticulata	>1000	>1000
Poales Poaceae Phyllostachys nigra	>1000	>1000
Poales Poaceae Phyllostachys viridiglaucescens	>1000	>1000
Poales Poaceae Phyllostachys vivax	>1000	>1000
Ranunculales Berberidaceae Berberis pruinosa	>1000	>1000
Ranunculales Berberidaceae Berberis oiwakensis	>1000	>1000
Cornales Cornaceae Cornus sanguinea	>1000	>1000
Ericales Theaceae Camellia japonica	>1000	>1000
Ericales Ericaceae Arbutus unedo	>1000	>1000
Ericales Actinidiaceae Actinidia chinensis	>1000	>1000
Ericales Balsaminaceae Impatiens balansae	>1000	>1000
Ericales Balsaminaceae Impatiens niamniamensis	>1000	>1000
Ericales Balsaminaceae Impatiens parviflora	>1000	>1000
Garryales Eucommiaceae Eucommia ulmoides	250	>1000
Asterales Asteraceae Centaurea nigra	>1000	>1000
Asterales Asteraceae Gymnanthemum amygdalinum	>1000	>1000
Asterales Asteraceae Silybum marianum	>1000	>1000
Asterales Asteraceae Helichrysum arwae	500	>1000
Asterales Asteraceae Achillea millefolium	>1000	>1000
Dipsacales Caprifoliaceae Valeriana officinalis	>1000	>1000
Dipsacales Viburnaceae Sambucus nigra	>1000	>1000
Dipsacales Viburnaceae Sambucus adnata	>1000	>1000
Apiales Apiaceae Bupleurum chinense var chinense	>1000	>1000
Apiales Apiaceae Astrantia major	>1000	>1000
Apiales Apiaceae Apium sellowianum	>1000	>1000
Lamiales Oleaceae Forsythia koreana	>1000	>1000
Lamiales Scrophulariaceae Scrophularia grandiflora	>1000	>1000
Lamiales Scrophulariaceae Verbascum spinosum	>1000	>1000
Lamiales Scrophulariaceae Verbascum nigrum subsp nigrum	>1000	>1000
Lamiales Scrophulariaceae Verbascum dumulosum	1000	>1000
Lamiales Acanthaceae Strobilanthes ixiocephala	>1000	>1000
Lamiales Bignoniaceae Newbouldia laevis	>1000	>1000
Lamiales Acanthaceae Odontonema tubaeforme	>1000	>1000
Lamiales Acanthaceae Justicia nyassan	1000	>1000
Lamiales Acanthaceae Aphelandra scabraa	250	>1000
Lamiales Acanthaceae Acanthus spinosus	>1000	>1000
Solanales Solanaceae Mellissia begoniifolia	>1000	>1000
Solanales Solanaceae Solanum laciniatum	>1000	>1000

Solanales Solanaceae Withania aristata	>1000	>1000
Solanales Solanaceae Withania riebeckii	>1000	>1000
Solanales Solanaceae Withania somnifera	>1000	>1000
Gentianales Apocynaceae Cynanchum viminale	>1000	>1000
Gentianales Apocynaceae Catharanthus roseus	>1000	>1000
Gentianales Rubiaceae Gardenia jasminoides	>1000	>1000
Gentianales Rubiaceae Thliphthisa purpurea	>1000	>1000
Gentianales Rubiaceae Galium aparine	>1000	>1000
Gentianales Rubiaceae Galium verum	>1000	>1000
Lamiales Lamiaceae Tinnea zambesiaca	1000	>1000
Lamiales Lamiaceae Thymus herba - barona	>1000	>1000
Lamiales Lamiaceae Thymus serpyllum	>1000	>1000
Lamiales Lamiaceae Thymus vulgaris	7.81	>1000
Lamiales Lamiaceae Teucrium chamaedrys	>1000	>1000
Lamiales Lamiaceae Teucrium asiaticum	>1000	>1000
Lamiales Lamiaceae Teucrium kotschyanum	>1000	>1000
Lamiales Lamiaceae Teucrium marum	>1000	>1000
Lamiales Lamiaceae Teucrium montanum	>1000	>1000
Lamiales Lamiaceae Teucrium orientale	>1000	>1000
Lamiales Lamiaceae Teucrium polium	>1000	>1000
Lamiales Lamiaceae Teucrium scorodonia	>1000	>1000
Lamiales Lamiaceae Teucrium subspinosum	>1000	>1000
Lamiales Lamiaceae Teucrium webbianum	>1000	>1000
Lamiales Lamiaceae Scutellaria albida	>1000	>1000
Lamiales Lamiaceae Scutellaria tomentosa	>1000	>1000
Lamiales Lamiaceae Scutellaria baicalensis	>1000	>1000
Lamiales Lamiaceae Scutellaria barbata	1000	>1000
Lamiales Lamiaceae Scutellaria diffusa	>1000	>1000
Lamiales Lamiaceae Scutellaria galericulata	>1000	>1000
Lamiales Lamiaceae Scutellaria tournefortii	>1000	>1000
Lamiales Lamiaceae Satureja montana	>1000	>1000
Lamiales Lamiaceae Salvia viridis	>1000	>1000
Lamiales Lamiaceae Salvia verticillata	>1000	>1000
Lamiales Lamiaceae Salvia involucrata	>1000	>1000
Lamiales Lamiaceae Salvia aethiopis	>1000	>1000
Lamiales Lamiaceae Salvia blancoana	>1000	>1000
Lamiales Lamiaceae Salvia candelabrum	>1000	>1000
Lamiales Lamiaceae Salvia divinorum	>1000	>1000
Lamiales Lamiaceae Salvia dorisiana	>1000	>1000
Lamiales Lamiaceae Salvia elegans	1000	>1000
Lamiales Lamiaceae Salvia gesneriflora	>1000	>1000
Lamiales Lamiaceae Salvia glutinosa	>1000	>1000
Lamiales Lamiaceae Salvia mexicana	>1000	>1000
Lamiales Lamiaceae Salvia officinalis	250	>1000
Lamiales Lamiaceae Salvia sclarea	>1000	>1000
Lamiales Lamiaceae Leonotis ocymifolia	>1000	>1000
Lamiales Lamiaceae Origanum vulgare	>1000	>1000
Lamiales Lamiaceae Isodon rugosus	500	>1000
Lamiales Lamiaceae Ocimum viphyense	>1000	>1000
Lamiales Lamiaceae Ocimum grandiflorum	62.5	>1000
Lamiales Lamiaceae Ocimum basilicum var basilicum	>1000	>1000
Lamiales Lamiaceae Ocimum gratissimum	>1000	>1000
Lamiales Lamiaceae Ocimum kilimandscharicum	>1000	>1000
Lamiales Lamiaceae Ocimum basilicum var minimum	>1000	>1000
Lamiales Lamiaceae Plectranthus ciliatus	1000	>1000
Lamiales Lamiaceae Plectranthus ecklonii	1000	>1000
Lamiales Lamiaceae Plectranthus elegans	125	>1000
Lamiales Lamiaceae Prunella vulgaris	>1000	>1000
Lamiales Lamiaceae Prostanthera cuneata	>1000	>1000
Lamiales Lamiaceae Coleus shirensis	>1000	>1000
Lamiales Lamiaceae Coleus paniculatus	500	>1000
Lamiales Lamiaceae Coleus crassus	125	>1000
Lamiales Lamiaceae Coleus hadiensis	1000	>1000
Lamiales Lamiaceae Coleus fruticosus	250	>1000
Lamiales Lamiaceae Coleus barbatus var grandis	1000	>1000
Lamiales Lamiaceae Coleus sp	500	>1000
Lamiales Lamiaceae Coleus hymalis	1000	>1000
Lamiales Lamiaceae Coleus burorum	>1000	>1000
Lamiales Lamiaceae Coleus coeruleus	>1000	>1000
Lamiales Lamiaceae Coleus	1000	>1000
Lamiales Lamiaceae Coleus cylindraceus	>1000	>1000
Lamiales Lamiaceae Eriope filifolia	1000	>1000

Lamiales Lamiaceae Eriope hypenioides	>1000	>1000
Lamiales Lamiaceae Eriope macrostachya	>1000	>1000
Lamiales Lamiaceae Collinsonia canadensis	>1000	>1000
Lamiales Phrymaceae Erythranthe cardinalis	>1000	>1000
Lamiales Paulowniaceae Paulownia fargesii	125	>1000
Lamiales Paulowniaceae Paulownia tomentosa	125	>1000
Lamiales Lamiaceae Cantinoa mutabilis	>1000	>1000
Lamiales Lamiaceae Pseudodictamnus mediterraneus	>1000	>1000
Lamiales Lamiaceae Asterohyptis stellulata	>1000	>1000
Lamiales Lamiaceae Ajuga reptans	>1000	>1000
Lamiales Lamiaceae Agastache rugosa	>1000	>1000
Lamiales Lamiaceae Agastache rupestris	>1000	>1000
Lamiales Lamiaceae Aeollanthus densiflorus	1000	>1000
Lamiales Lamiaceae Aeollanthus buchnerianus	1000	>1000
Lamiales Lamiaceae Betonica macrantha	>1000	>1000
Lamiales Lamiaceae Betonica officinalis	>1000	>1000
Lamiales Lamiaceae Stachys byzantina	>1000	>1000
Lamiales Lamiaceae Stachys germanica	>1000	>1000
Lamiales Lamiaceae Nepeta faassenii	>1000	>1000
Lamiales Lamiaceae Nepeta menthoides	>1000	>1000
Lamiales Lamiaceae Nepeta sibirica	>1000	>1000
Lamiales Lamiaceae Mentha longifolia	>1000	>1000
Lamiales Lamiaceae Mentha spicata	>1000	>1000
Lamiales Lamiaceae Mentha villosa	>1000	>1000
Lamiales Lamiaceae Marrubium cylleneum	>1000	>1000
Lamiales Lamiaceae Dracocephalum stamineum	>1000	>1000
Lamiales Lamiaceae Lamium flexuosum	>1000	>1000
Lamiales Lamiaceae Lamium galeobdolon	>1000	>1000
Lamiales Lamiaceae Lamium maculatum	>1000	>1000
Caryophyllales Portulacaceae Portulaca oleracea	>1000	>1000
Caryophyllales Caryophyllaceae Silene caryophylloides subsp echinus	1000	>1000
Caryophyllales Caryophyllaceae Silene coronaria	>1000	>1000
Caryophyllales Caryophyllaceae Silene spergulifolia	>1000	>1000
Caryophyllales Polygonaceae Persicaria capitata	500	>1000
Caryophyllales Polygonaceae Rumex rupestris	>1000	>1000
Caryophyllales Droseraceae Drosera macrantha subsp macrantha	500	1000
Caryophyllales Droseraceae Drosera aliciae	125	>1000
Caryophyllales Droseraceae Drosera capensis	>1000	>1000
Caryophyllales Droseraceae Drosera venusta	>1000	>1000
Caryophyllales Droseraceae Drosera dichrosepala	500	>1000
Caryophyllales Droseraceae Drosera binata	1000	>1000
Caryophyllales Droseraceae Drosera gibsonii	125	1000
Caryophyllales Droseraceae Drosera platystigma	500	1000
Caryophyllales Droseraceae Drosera affinis	500	>1000
Vitales Vitaceae Leea angulata	>1000	>1000
Saxifragales Saxifragaceae Saxifraga spathularis	>1000	>1000
Saxifragales Grossulariaceae Ribes speciosum	>1000	>1000
Saxifragales Paeoniaceae Paeonia lactiflora	>1000	>1000
Saxifragales Paeoniaceae Paeonia ostii	>1000	>1000
Saxifragales Crassulaceae Crassula brevifolia subsp Brevifolia	1000	>1000
Myrtales Combretaceae Terminalia sericea	>1000	>1000
Myrtales Lythraceae Punica granatum	>1000	>1000
Myrtales Myrtaceae Melaleuca citrina	>1000	>1000
Myrtales Myrtaceae Melaleuca rhaphiophylla	>1000	>1000
Myrtales Myrtaceae Leptospermum continentale	>1000	>1000
Myrtales Myrtaceae Leptospermum lanigerum	>1000	>1000
Myrtales Myrtaceae Kunzea ericoides	>1000	>1000
Myrtales Myrtaceae Eucalyptus aggregata	62.5	>1000
Myrtales Myrtaceae Eucalyptus chapmaniana	250	>1000
Myrtales Myrtaceae Eucalyptus dalrympleana	500	>1000
Myrtales Myrtaceae Eucalyptus gunnii	31.3	>1000
Myrtales Myrtaceae Eucalyptus pauciflora	125	>1000
Myrtales Myrtaceae Eucalyptus perriniana	250	>1000
Geraniales Geraniaceae Pelargonium crispum	>1000	>1000
Geraniales Geraniaceae Geranium phaeum	>1000	>1000
Geraniales Geraniaceae Geranium kotschyi	>1000	>1000
Malpighiales Malpighiaceae Banisteriopsis caapi	>1000	>1000
Malpighiales Phyllanthaceae Phyllanthus casticum	>1000	>1000
Malpighiales Phyllanthaceae Phyllanthus emblica	>1000	>1000

Malpighiales Passifloraceae Turnera ulmifolia	>1000	>1000
Malpighiales Euphorbiaceae Omphalea hypoleuca	>1000	>1000
Malpighiales Euphorbiaceae Euphorbia pulcherrima	1000	>1000
Malpighiales Euphorbiaceae Croton trinitatis	>1000	>1000
Malpighiales Clusiaceae Garcinia livingstonei	1000	>1000
Malpighiales Hypericaceae Hypericum addingtonii	>1000	>1000
Malpighiales Hypericaceae Hypericum bellum	>1000	>1000
Malpighiales Hypericaceae Hypericum forrestii	>1000	>1000
Malpighiales Hypericaceae Hypericum henryi subsp henryi	500	>1000
Malpighiales Hypericaceae Hypericum hircinum	>1000	>1000
Malpighiales Hypericaceae Hypericum hookerianum	>1000	>1000
Malpighiales Hypericaceae Hypericum latisepalum	>1000	>1000
Malpighiales Hypericaceae Hypericum stellatum	>1000	>1000
Sapindales Rutaceae Zanthoxylum heterophyllum	>1000	>1000
Sapindales Burseraceae Commiphora dulcis	>1000	>1000
Sapindales Anacardiaceae Dracontomelon dao	62.5	>1000
Brassicales Brassicaceae Berteroa mutabilis	>1000	>1000
Brassicales Brassicaceae Crambe maritima	>1000	>1000
Malvales Thymelaeaceae Daphne tangutica	1000	500
Malvales Thymelaeaceae Daphne pontica	1000	>1000
Malvales Thymelaeaceae Daphne laureola	500	>1000
Malvales Thymelaeaceae Daphne gemmata	>1000	>1000
Malvales Thymelaeaceae Daphne bholua	1000	>1000
Malvales Thymelaeaceae Daphne alpina	>1000	1000
Malvales Cistaceae Helianthemum cinereum subsp cinereum	>1000	>1000
Malvales Malvaceae Abutilon grandifolium	>1000	>1000
Malvales Malvaceae Sterculia mexicana	1000	>1000
Malvales Malvaceae Tilia euchlora	1000	>1000
Malvales Malvaceae Tilia platyphyllos subsp corinthiaca	>1000	>1000
Malvales Malvaceae Tilia tomentosa	>1000	>1000
Malvales Malvaceae Tilia europaea	>1000	>1000
Fagales Betulaceae Betula pendula	>1000	>1000
Fagales Betulaceae Betula pendula subsp pendula	>1000	>1000
Fagales Fagaceae Quercus infectoria	>1000	1000
Fagales Fagaceae Castanea sativa	>1000	>1000
Fabales Fabaceae Pueraria montana var lobata	>1000	>1000
Fabales Fabaceae Pongamia pinnata	>1000	>1000
Fabales Fabaceae Peltophorum dubium	>1000	>1000
Fabales Fabaceae Lupinus arboreus	1000	>1000
Fabales Fabaceae Lonchocarpus costaricensis	>1000	>1000
Fabales Fabaceae Erythrina lysistemon	31.3	>1000
Fabales Polygalaceae Chamaebuxus alpestris	>1000	>1000
Fabales Fabaceae Staminodianthus duckei	>1000	>1000
Fabales Fabaceae Staminodianthus racemosus	>1000	>1000
Fabales Fabaceae Staminodianthus rosae	>1000	>1000
Fabales Fabaceae Sophora flavescens var flavescens	>1000	>1000
Fabales Fabaceae Sophora flavescens	>1000	>1000
Fabales Fabaceae Sophora fulvida	>1000	>1000
Fabales Fabaceae Sophora microphylla	>1000	>1000
Fabales Fabaceae Sophora tetraptera	>1000	>1000
Cucurbitales Cucurbitaceae Bryonia alba	>1000	>1000
Rosales Rosaceae Crataegus persimilis	>1000	>1000
Rosales Rosaceae Filipendula vulgaris	>1000	>1000
Rosales Rosaceae Geum coccineum	>1000	>1000
Rosales Rosaceae Sorbus aucuparia	>1000	>1000
Rosales Rosaceae Hedlundia takhtajanii	>1000	>1000
Rosales Moraceae Filipendula ulmaria	>1000	>1000
Rosales Moraceae Morus alba	>1000	>1000
Rosales Moraceae Artocarpus heterophyllus	>1000	>1000
Rosales Moraceae Maclura pomifera	500	>1000
Rosales Moraceae Ficus religiosa	>1000	>1000
Rosales Moraceae Ficus thonningii	>1000	>1000
Rosales Moraceae Ficus lyrata	250	>1000
Rosales Moraceae Ficus lingua	125	>1000
Rosales Moraceae Ficus coronata	>1000	>1000
Rosales Moraceae Ficus cordata	>1000	>1000
Rosales Moraceae Ficus carica	>1000	>1000
Rosales Moraceae Ficus benghalensis	>1000	>1000
Rosales Moraceae Dorstenia arifolia	>1000	>1000
Rosales Moraceae Dorstenia bahiensis	>1000	>1000

Rosales Moraceae Dorstenia barteri	15.6	>1000
Rosales Moraceae Dorstenia barteri var multiradiata	62.5	>1000
Rosales Moraceae Dorstenia brownii	>1000	>1000
Rosales Moraceae Dorstenia christenhuszii	>1000	>1000
Rosales Moraceae Dorstenia contrajerva	>1000	>1000
Rosales Moraceae Dorstenia foetida	>1000	>1000
Rosales Moraceae Dorstenia foetida subsp lancifolia	>1000	>1000
Rosales Moraceae Dorstenia gigas	>1000	>1000
Rosales Moraceae Dorstenia hildebrandtii	>1000	>1000
Rosales Moraceae Dorstenia psilurus	1000	>1000

Table S3. MIC values of serial extracts vs SA

				Hexane	EtOAc	60%MeOH
BI Number	Family	Genus	Species	MIC	MIC	MIC
3210	Lamiaceae	Tinnea	zambesiaca	>1000	>1000	1000
29881	Malvaceae	Tilia	euchlora	500	125	1000
33234	Lamiaceae	Solenostemon	manii	>1000	>1000	125
33369	Lamiaceae	Salvia	sclarea	250	>1000	>1000
33885	Lamiaceae	Salvia	elegans	62.5	500	500
31525	Lamiaceae	Salvia	officialis	>1000	250	125
1694	Lamiaceae	Rabdosia	rugova	>1000	62.5	>1000
28494	Fagaceae	Quercus	infectoria	>1000	>1000	>1000
33775	Lythraceae	Punica	granatum	>1000	>1000	1000
33218	Lamiaceae	Plecthrantus	fruticosus	62.5	500	>1000
33220	Lamiaceae	Plecthrantus	cyaneus	500	250	>1000
33221	Lamiaceae	Plecthrantus	elegans	15.6	15.6	1000
33222	Lamiaceae	Plecthrantus	ecklonii	>1000	125	1000
33224	Lamiaceae	Plecthrantus	grandis	>1000	500	>1000
33225	Lamiaceae	Plecthrantus	hadiensis	62.5	7.8	>1000
33226	Lamiaceae	Plecthrantus	hyemalis	500	125	>1000
33227	Lamiaceae	Plecthrantus	coleoides	7.8	1.95	>1000
1671	Lamiaceae	Plecthrantus	crassus	7.8	1.95	62.5
33229	Lamiaceae	Plecthrantus	ciliatus	>1000	>1000	>1000
33847	Fabaceae	Peltophorum	dubium	>1000	>1000	>1000
33565	Paulowniaceae	Paulownia	fargesii	125	31.3	250
29680	Phrymaceae	Mimulus	cardinalis	>1000	>1000	>1000
33368	Berberidaceae	Mahonia	lomariifolia	>1000	>1000	>1000
31757	Moraceae	Maclura	pomifera	7.8	3.9	500
33641	Fabaceae	Lupinus	arboreus	500	62.5	>1000
33642	Fabaceae	Lupinus	arboreus	31.3	500	>1000
29098	Fabaceae	Lonchocarpus	costaricensis	15.6	62.5	>1000
32008	Hypericaceae	Hypericum	forestii	>1000	>1000	125
32010	Asteraceae	Helichrysum	arwae	31.3	62.5	500
33717	Clusiaceae	Garcinia	livingstone	>1000	>1000	>1000
	Moraceae	Ficus	coronata	>1000	>1000	>1000
33722	Euphorbiaceae	Euphorbia	pulcherrima	>1000	>1000	>1000
19484	Eucommiaceae	Eucomnia	ulmoides	>1000	>1000	1000
33564	Myrtaceae	Eucalyptus	gunnii	>1000	31.3	>1000
33370	Myrtaceae	Eucalyptus	dalrympleana	7.8	15.6	1000
30985	Myrtaceae	Eucalyptus	perriniana	>1000	62.5	250
30980	Myrtaceae	Eucalyptus	aggregata	>1000	31.3	62.5
31852	Myrtaceae	Eucalyptus	pauciflora	125	31.3	250
30986	Myrtaceae	Eucalyptus	chapmaniana	>1000	62.5	250
34569	Droseraceae	Drosera	gibsonii	250	>1000	1000
34573	Droseraceae	Drosera	binata	125	250	>1000
34575	Droseraceae	Drosera	dichrosepala	>1000	31.3	1000
34535	Droseraceae	Drosera	venusta	250	3.9	>1000
34636	Droseraceae	Drosera	capensis	500	15.6	>1000
34639	Droseraceae	Drosera	aliciae	500	3.9	>1000
34640	Droseraceae	Drosera	macrantha subsp. Macrantha	500	500	>1000
34459	Droseraceae	Drosera	affinis	250	62.5	31.3
34463	Droseraceae	Drosera	platystigma	250	500	500
33845	Anacardiaceae	Dracontomelon	dao	15.6	>1000	>1000
33725	Fabaceae	Derris	trifoliata	>1000	>1000	>1000
33646	Thymelaeaceae	Daphne	bholua	125	125	1000
33648	Thymelaeaceae	Daphne	laureola	125	125	>1000
33927	Thymelaeaceae	Daphne	laureola	>1000	>1000	>1000

1829	Lamiaceae	Coleus	kilimandschariens	125	125	1000
31178	Berberidaceae	Berberis	pruinosa	>1000	500	>1000
1645	Lamiaceae	Becium	grandiflora	500	31.3	>1000
33230	Lamiaceae	Aeollanthus	buchniorianus	125	250	>1000
33231	Lamiaceae	Aeollanthus	densiflorus	62.5	31.3	>1000
2732	Lamiaceae	Aeollanthus	njasse	250	1000	1000

Table S4. MIC values of serial extracts vs EC

BI Number	Family	Genus	Species	Hexane	EtOAc	60%MeOH	EtOH
				MIC	MIC	MIC	MIC
2732	Lamiaceae	Aeollanthus	njasse	>1000	>1000	>1000	
3210	Lamiaceae	Tinnea	zambesiaca	>1000	>1000	>1000	
33218	Lamiaceae	Plecthrantus	fruticosus	>1000	>1000	>1000	
33220	Lamiaceae	Plecthrantus	cyaneus	>1000	>1000	>1000	
33221	Lamiaceae	Plecthrantus	elegans	>1000	>1000	>1000	
33222	Lamiaceae	Plecthrantus	ecklonii	>1000	>1000	>1000	
33224	Lamiaceae	Plecthrantus	grandis	>1000	>1000	>1000	
33225	Lamiaceae	Plecthrantus	hadiensis	>1000	>1000	>1000	
33226	Lamiaceae	Plecthrantus	hyemalis	>1000	>1000	>1000	
33227	Lamiaceae	Plecthrantus	coleoides	>1000	>1000	>1000	
1645	Lamiaceae	Becium	grandiflora	>1000	>1000	>1000	
1829	Lamiaceae	Coleus	kilimandschariens	>1000	>1000	>1000	
1694	Lamiaceae	Rabdosia	rugova	>1000	>1000	>1000	
1671	Lamiaceae	Plecthrantus	crassus	>1000	>1000	>1000	
33229	Lamiaceae	Plecthrantus	ciliatus	>1000	>1000	>1000	
33230	Lamiaceae	Aeollanthus	buchniorianus	>1000	>1000	>1000	
33231	Lamiaceae	Aeollanthus	densiflorus	>1000	>1000	>1000	
33234	Lamiaceae	Solenostemon	manii	>1000	>1000	>1000	
33641	Fabaceae	Lupinus	arboreus	>1000	>1000	>1000	
33642	Fabaceae	Lupinus	arboreus	>1000	>1000	>1000	
33646	Thymelaeaceae	Daphne	bholua	>1000	>1000	>1000	
33648	Thymelaeaceae	Daphne	laureola	>1000	>1000	>1000	
33717	Clusiaceae	Garcinia	livingstone	>1000	>1000	>1000	
33722	Euphorbiaceae	Euphorbia	pulcherrima	>1000	>1000	>1000	
33725	Fabaceae	Derris	trifoliata	>1000	>1000	>1000	
33564	Myrtaceae	Eucalyptus	gunnii	>1000	>1000	>1000	
33370	Myrtaceae	Eucalyptus	dalrympleana	>1000	>1000	>1000	
33368	Berberidaceae	Mahonia	lomariifolia	>1000	>1000	>1000	
33369	Lamiaceae	Salvia	sclarea	>1000	>1000	>1000	
33845	Anacardiaceae	Dracontomelon	dao	>1000	>1000	>1000	
33775	Lythraceae	Punica	granatum	>1000	>1000	>1000	
33565	Paulowniaceae	Paulownia	fargesii	>1000	>1000	>1000	
33927	Thymelaeaceae	Daphne	laureola	>1000	1000	>1000	
29881	Malvaceae	Tilia	euchlora	>1000	>1000	1000	
19484	Eucommiaceae	Eucomnia	ulmoides	>1000	>1000	>1000	
33885	Lamiaceae	Salvia	elegans	>1000	>1000	>1000	
31757	Moraceae	Maclura	pomifera	>1000	>1000	>1000	
33847	Fabaceae	Peltophorum	dubium	>1000	>1000	>1000	
28494	Fagaceae	Quercus	infectoria	>1000	>1000	>1000	
	Moraceae	Ficus	coronata	>1000	>1000	>1000	
31852	Myrtaceae	Eucalyptus	pauciflora	>1000	>1000	>1000	
32010	Asteraceae	Helichrysum	arwae	>1000	>1000	>1000	
32008	Hypericaceae	Hypericum	forestii	>1000	>1000	>1000	

29680	Phrymaceae	Mimulus	cardinalis	>1000	>1000	>1000	
30985	Myrtaceae	Eucalyptus	perriniana	>1000	>1000	>1000	
29098	Fabaceae	Lonchocarpus	costaricensis	>1000	>1000	>1000	
30986	Myrtaceae	Eucalyptus	chapmaniana	>1000	>1000	>1000	
31178	Berberidaceae	Berberis	pruinosa	>1000	>1000	>1000	
31525	Lamiaceae	Salvia	officialis	>1000	>1000	>1000	
30980	Myrtaceae	Eucalyptus	aggregata	>1000	>1000	>1000	
	**BI Number**	**Genus**	**Species**				
34569	34569	Drosera	gibsonii	1000	>1000	>1000	1000
34573	34573	Drosera	binata	125	>1000	>1000	>1000
34575	34575	Drosera	dichrosepala	>1000	>1000	>1000	>1000
34535	34535	Drosera	venusta	>1000	>1000	>1000	>1000
34636	34636	Drosera	capensis	>1000	1000	>1000	>1000
34639	34639	Drosera	aliciae	>1000	125	>1000	>1000
34640	34640	Drosera	macrantha subsp. Macrantha	>1000	250	>1000	1000
34459	34459	Drosera	affinis	>1000	>1000	>1000	>1000
34463	34463	Drosera	platystigma	1000	>1000	>1000	1000
		Daphne	gemmata				>1000
		Daphne	gemmata				>1000
		Daphne	alpina				1000
		Daphne	alpina				1000
		Daphne	tangutica				500
		Daphne	tangutica				1000
